# Online moral deviance: an integrative review of digital behaviors

**DOI:** 10.3389/fpsyg.2025.1573164

**Published:** 2025-07-24

**Authors:** Xu Chen, Norzihani Saharuddin, Maizura Yasin, Meng Wang

**Affiliations:** Faculty of Educational Studies, Universiti Putra Malaysia, Serdang, Selangor, Malaysia

**Keywords:** deviant behaviors, digital environments, cyber deviance, conceptual model, online moral deviance

## Abstract

Previous studies on deviant behaviors in digital environments have predominantly focused on the concept of cyber deviance, online deviance and online deviant behavior, a broad behavioral framework encompassing diverse and varied actions. However, existing research has not systematically classified these behaviors based on their characteristics, manifestations or the degree of harm caused to target users within a moral framework. To addresses this research gap, our study systematically identifies and classifies behaviors within cyber deviance, online deviance and online deviant behavior that violate moral expectations accepted by mainstream culture in a specific society, and develops a conceptual model of online moral deviance. The proposed model provides valuable insights for effectively identifying, preventing, and addressing such deviant behaviors in digital contexts. Utilizing an integrative literature review approach, we analyzed research from the Web of Science (2020–2024) database and Google Scholar, employing core, combined, and related keywords to identify relevant studies. A total of 190 articles were selected, including key research from the past 5 years and representative studies beyond this timeframe. Moreover, our research emphasizes the importance of considering cultural, regional, and social contextual differences in refining the conceptual model. This study advocates for future research to explore representative theories that can support the mechanisms or influencing factors underlying online moral deviance, and to refine the conceptual model by clearly delineating the conceptual boundaries between different types of online deviant behavior. This study advances the research on cyber deviance and online deviance, and, in particular, offers a practical conceptual model for policymakers, educators, and parents to support moral education and foster healthy online behavior.

## 1 Introduction

The deep application of the Internet has created opportunities for both individuals and industries, but it has also raised ethical concerns. For instance, Peters et al. (2021) demonstrate that video ethics has emerged as a recent topic in educational research. Armond et al. (2021) highlight the increasing focus of academics on research ethics. Huang et al. (2023) stated that the extensive integration of AI into social and economic domains is bound to influence broad areas of society and lead to the emergence of ethical issues related to AI. It is evident that ethical issues have become critical topics in the study of digital behaviors. This has prompted stakeholders to prioritize ethics-focused research and respond accordingly (Kazim and Koshiyama, 2021).

Erikson (1962) proposed that deviance refers to behaviors generally recognized as requiring the attention of social control institutions. Deviance is characterized by violations of moral norms and established rules in mainstream society (Parsons, 1951). Sociological findings suggest that an actors' online deviant behavior and offline deviant behavior may translate into each other (Plé and Demangeot, 2020). In fact, some deviant behaviors have shifted from offline to online environment, new deviant behaviors may also arise entirely in the online environment. These deviant behaviors that occur in the online environment are known as cyber deviance. The new practices of cyber deviance, such as Internet trolling, online flaming, and hacking are occurring more frequently (Udris, 2017). Studies have shown that these unethical digital behaviors can have a negative impact on victims. For instance, Wilson and Seigfried-Spellar (2023) demonstrated that internet trolling can lead to psychological harm among its victims. The findings of Ossa et al. (2023) indicate that cyberbullying exhibits specific associations with mental health, risk-taking behaviors, and self-injurious actions among targeted individuals. The findings of Li et al. (2019) suggest that, compared to non-victimized individuals, cyber victims exhibit greater prevalence of sleep disorders and physical discomfort. Relevant research evidence underscores the necessity and urgency to focus on and study whether individual or group behaviors in online environments have violated the moral expectations widely accepted within the mainstream culture of a given society.

Previous research has shown that the research topic most closely associated with online moral deviance are cyber deviance, online deviance and online deviant behavior. Based on a thorough review, analysis, and synthesis of the existing literature on those topic of cyber deviance, online deviance and online deviant behavior, We have identified the following research gaps: First, in existing studies centered on *cyber deviance, online deviance*, and *online deviant behavior*, a wide range of digital behaviors—including digital/media piracy, illegal website visits, online flaming, internet pornography (Chen et al., 2021), online sexual harassment, cyberbullying, hacking (Zhou et al., 2024), internet rumors, deception on the internet (Jin et al., 2022), cyber dating abuse, and social spamming (Cioban et al., 2021)—have been broadly grouped under these umbrella terms. The spectrum of digital behaviors that fall under *cyber deviance, online deviance*, or *online deviant behavior* is extensive. In fact, these digital behaviors span both moral and legal domains. However, under the thematic focus of *cyber deviance, online deviance*, and *online deviant behavior*, existing literature does not distinguish these behaviors from either a moral or legal perspective, nor does it examine them separately through these two lenses. Second, existing research on *cyber deviance, online deviance*, and *online deviant behavior* predominantly focuses on specific types of digital behavior within these overarching themes. Based on our systematic analysis of the existing literature on the above three research themes, most studies tend to concentrate on only one behavioral type—such as internet plagiarism (e.g., Eret and Ok, 2014), online trolling (e.g., Thacker and Griffiths, 2012) or online cheating (e.g., Noorbehbahani et al., 2022). No study have specifically focused on integrating these individual digital behaviors from a moral framework perspective. Researchers have demonstrated a preference for examining specific phenomena rather than adopting an integrated approach to these behavioral patterns (Udris, 2017).

To address the above research gaps, it is necessary to identify, classify, and integrate different categories of digital behaviors from a moral framework perspective within the broader research themes of cyber deviance, online deviance, and online deviant behavior, and to construct a conceptual model of these behaviors based on that moral framework. The development of the conceptual model of online moral deviance contributes to deepening research on cyber deviance, online deviance, and online deviant behavior. This conceptual model also offers practical value for policymakers, educators, and parents. First, the model can help policymakers, educators, and parents develop a deeper understanding of this emerging behavioral pattern—online moral deviance. In addition, the model provides a foundation for policymakers to design more targeted moral education policies in response to digital behaviors, thereby helping to regulate online conduct, especially among adolescents and other vulnerable groups. Lastly, particularly in the fields of school and family education, the proposed conceptual model can inspire educators and parents to analyze the factors influencing this behavioral pattern. It also supports schools in enhancing curriculum design related to moral education and digital life, and it helps both schools and parents to reflect on effective preventive strategies for addressing this type of behavior.

Depending on the methodology required to achieve the purpose of the review, a literature review may be the most appropriate methodological tool when it serves as a foundation for developing a new conceptual model or theory (Snyder, 2019). The purpose of an integrative review is to evaluate, critique, and synthesize literature on a specific research topic (Torraco, 2005). Its search strategy is usually not systematic, and it often integrates literature using taxonomy or classification approaches (Snyder, 2019). Moreover, it should not be merely descriptive or historical, but should preferably generate a new conceptual framework or theory (Snyder, 2019). Considering that the aim of our study is to identify, categorize, and synthesize digital behaviors across different disciplines that violate widely accepted moral expectations within a specific society's dominant culture—and to construct a conceptual model of such behavioral patterns through classification and integration—an integrative review is an appropriate review method, as it aligns with both the purpose and characteristics of this type of review.

## 2 Method

### 2.1 Database selection and theoretical rationale

Our study utilized both the Web of Science database and Google Scholar for literature retrieval. Web of Science was selected as the primary database due to its inclusion of high-impact, peer-reviewed journals across multiple disciplines, particularly in behavioral sciences, psychology, education, sociology, and criminology—fields directly aligned with online moral deviance research. Based on the literature retrieval results from the Web of Science database, we further employed Google Scholar for literature searching. This decision was made because analysis of the literature retrieved from Web of Science revealed that the number of highly relevant articles included in Web of Science over the past 5 years was limited. Most critically, these retrieved articles failed to systematically explain why certain online deviant behaviors can be considered as components of online moral deviance. Given that Google Scholar has extensive literature coverage and includes earlier representative works, and considering that our research objective is to identify representative literature that can explain why certain digital behaviors can be considered as components of a conceptual model of online moral deviance, we therefore further employed Google Scholar for literature retrieval.

### 2.2 Inclusion and exclusion criteria

To enhance methodological rigor and transparency, predefined inclusion and exclusion criteria were established:

Inclusion criteria were: ① Topical relevance: focus on digital behaviors that violate widely accepted moral expectations within a particular mainstream culture (e.g., online plagiarism, online cheating, flaming); ② Conceptual or empirical contribution: articles that provided definitions, typologies, consequences, concrete manifestations, or moral/ethical interpretations of such deviant behaviors; ③ Peer-reviewed: inclusion of original research articles or review articles published in peer-reviewed journals; ④ Language and time frame: English literature published between 2020 and 2024 in Web of Science; broader time frame allowed in Google Scholar to identify representative literature.

Exclusion criteria included: ① Articles with no explicit moral or ethical framing—articles that focused solely on online deviant behaviors without reference to moral, or ethical concerns. Studies involving online deviant behaviors related to cybercrime (e.g., hacking, online fraud, digital piracy) rather than a moral lens; ② Academic format (excluding commentaries, conference proceedings, editorials, and non-peer-reviewed publications); ③ Duplicate entries.

### 2.3 Search strategy and keyword design

First, core keywords and combined keywords were employed to search Web of Science for English-language peer-reviewed literature published between 2020 and 2024, to identify the most recent research exploring online deviance, cyber deviance, and online deviant behavior within moral frameworks over the past 5 years. Second, we systematically analyzed the literature retrieved with these core keywords and combined keywords to identify the major categories of online deviant behaviors (e.g., online plagiarism, flaming, online cheating) that constitute the conceptual model of online moral deviance. The identified major categories of online deviant behaviors form the core components of the conceptual framework of online moral deviance, as these online deviant behaviors violate widely accepted moral expectations within specific mainstream cultural contexts in given society. Based on the above analysis, we identified the relevant keywords used for literature retrieval in Web of Science. These relevant keywords represent specific types of online deviant behavior that constitute online moral deviance, as they are considered components of online moral deviance or elements of the conceptual framework of online moral deviance.

The core keywords employed in Web of Science included online deviance, cyber deviance, and online deviant behavior, terms that are considered conceptually similar in existing research. Literature retrieval utilized Boolean logic combinations: online deviance OR cyber deviance OR online deviant behavior. The combined keywords used in Web of Science involved Boolean logic combinations of terms includes cyber deviance, online deviance, online deviant behavior, digital behavior, and moral, aimed at achieving more comprehensive coverage of literature relevant to this research topic. These core keywords and combined keywords ensure high relevance of selected literature to the research theme of online moral deviance. The related keywords used in Web of Science encompassed Internet plagiarism or online plagiarism, Internet trolling or online trolling, cyberbullying, online harassment or Internet harassment, academic misconduct, online cheating, and flaming. Given the substantial volume of literature on cyberbullying in Web of Science, and our research focuses on the concepts, nature, and characteristics of digital behaviors, our study employed a targeted approach to retrieve research that is closely aligned with the topic under investigation. The core search categories included psychology multidisciplinary, psychology developmental, psychology social, education educational research, sociology, communication, family studies, and criminology penology. This focus ensured comprehensive coverage across key disciplines such as education, psychology, sociology, communication studies, and criminology.

After applying the above strategy to identify relevant keywords and conducting searches in the Web of Science, it was found that the number of articles highly relevant to the research topic was limited. The literature identified through Web of Science did not provide a systematic explanation of why the identified categories of online deviant behaviors could be considered components of online moral deviance. Considering that Google Scholar indexes a broader range of sources and includes earlier representative works, we therefore chose to use Google Scholar to retrieve representative literature involving the relevant keywords.

This means that the keywords used to search the literature in Google Scholar were derived from the relevant keywords applied in the Web of Science. In other words, all keywords used in Google Scholar were identical to the related keywords used in Web of Science. The use of related keywords determined in Web of Science for literature retrieval in Google Scholar aligns with our research objectives. This is because our research purpose is to explain why certain digital behaviors can be considered as components of online moral deviance or as constituent elements of the conceptual framework of online moral deviance. This means that our use of Google Scholar was not intended to retrieve all literature explaining why certain type of online deviant behaviors can constitute online moral deviance, but rather to locate representative literature. As Snyder (2019) noted in a high-quality study on literature review methodologies, “this type of review often requires a more creative collection of data.” The purpose of an integrative review is not usually to cover all the articles ever published on the topic but rather to combine perspectives and insights from different fields or research traditions (Snyder, 2019).

It was precisely because the representative literature retrieved from Web of Science using the relevant keywords was insufficient and could not meet our research objectives that we further chose to use Google Scholar to retrieve only the representative literature related to those related keywords. Based on the above rationale, the keywords for literature retrieval in Google Scholar included Internet plagiarism, online plagiarism, Internet trolling, online trolling, cyberbullying, online harassment, Internet harassment, academic misconduct, online cheating, and flaming. Considering that representative research findings on specific categories of online deviant behaviors as components of online moral deviance may extend beyond the five-year timeframe, the temporal scope of English-language peer-reviewed literature retrieved through Google Scholar was not restricted to within 5 years.

### 2.4 The process, criteria, and results of literature screening

In the WOS database, we selected literature for inclusion in the final sample following these steps and criteria: first, we retrieved literature using core keywords and combined keywords in WOS and read the titles and abstracts of these articles. The literature evaluation criterion for this step was topical relevance, meaning that the literature focused on online deviant behaviors that violate widely accepted moral expectations within specific mainstream cultural contexts. Second, we further conducted full-text reading of the literature screened through the first step, selecting articles that aligned with the research objectives. The literature evaluation criterion for this step was alignment with research objectives, namely these studies investigate the definition, nature, and specific manifestations of one or multiple types of online deviant behaviors that violate widely accepted moral expectations within a particular mainstream culture, or examine the physical, psychological, or emotional harm that certain online deviant behaviors inflict upon targeted network users. These studies provide supportive explanations for why certain online deviant behaviors can constitute a behavioral pattern of online moral deviance or can be incorporated into the conceptual framework of online moral deviance.

The specific process we used for searching literature on Google Scholar was as follows: first, we identified relevant keywords through Web of Science and used those keywords to search for literature in Google Scholar. Second, the literature retrieved in Google Scholar using those keywords was vast. Considering that the purpose of an integrative review is to synthesize perspectives and insights from different fields—not to cover all literature related to the topic—we further filtered the literature by prioritizing highly cited papers or those indexed in SSCI/SCI, which improved the representativeness and credibility of the selected articles. We further determined which literature among highly cited articles or SSCI/SCI-indexed publications could be included as literature for the final sample by reading titles and abstracts. The literature evaluation criterion for Google Scholar was also alignment with research objectives.

**Figure 1:**
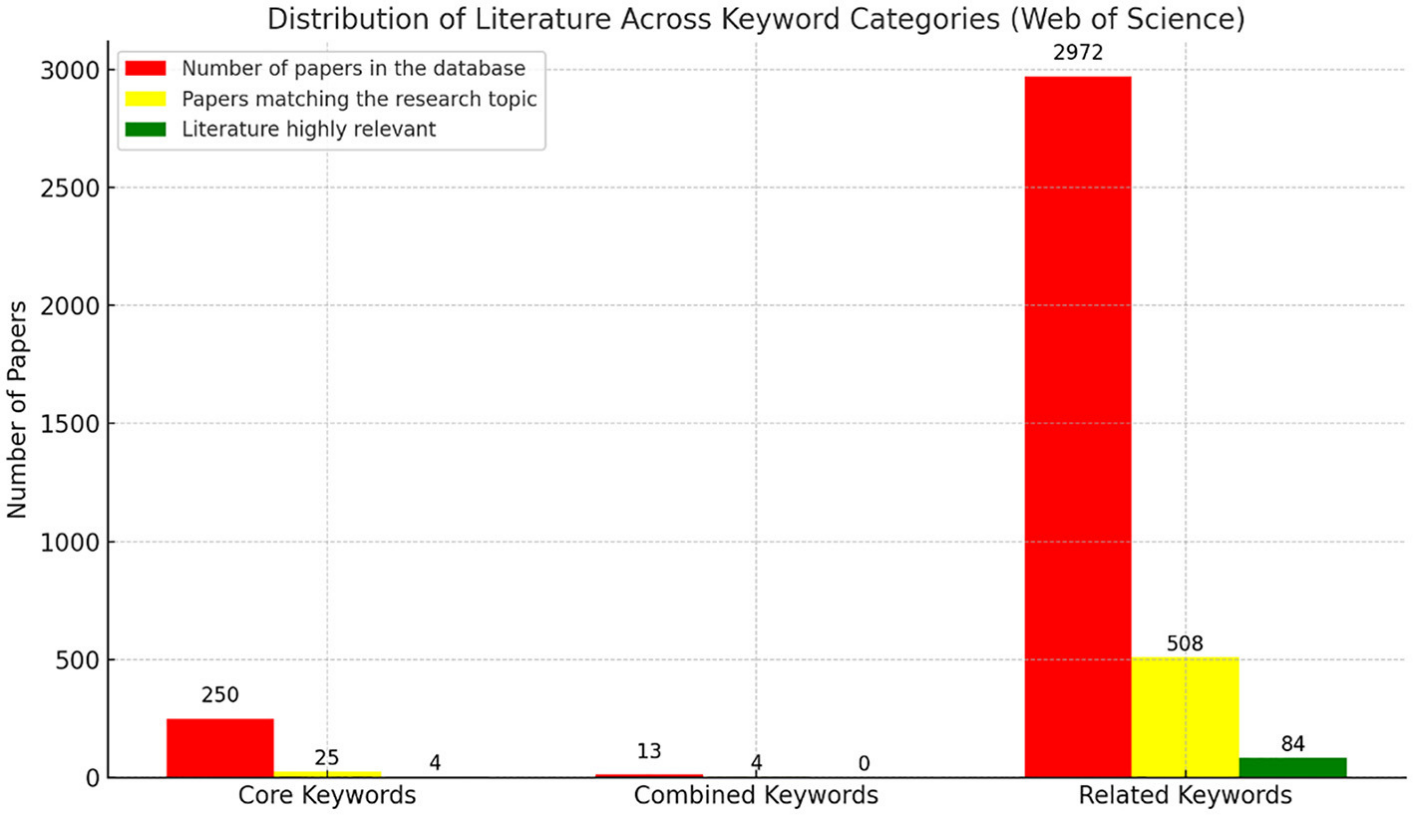
Distribution of literature across keyword categories (web of science).

Following the outlined search procedure, 250 papers were retrieved from the Web of Science database using the core keywords. From these, 25 papers were identified as relevant to the research topic, and ultimately, 4 papers were deemed highly relevant. Using the combined keywords in the Web of Science database, 13 papers were initially retrieved, with 4 identified as relevant to the research topic, though none were classified as highly relevant. When applying the related keywords, 2,972 papers were retrieved, 508 of which were relevant, and 84 were categorized as highly relevant to the study. In total, 88 articles highly relevant to the research topic were identified within the Web of Science database. Using related keywords in Google Scholar, a total of 105 highly cited articles and peer-reviewed journal articles indexed in SSCI or SCI were retrieved. Through evaluating the alignment of these articles with research objectives, 102 articles were ultimately determined for inclusion in the final sample. In total, this study obtained 190 reference articles. Figure 1 below shows an overview of the literature search results conducted using the Web of Science database, and detailed findings displayed in Table 1 below.

**Table 1:** Summary of literature search results from the Web of Science database using core, combined, and related keywords.

**Data base: Web of Science**			
**Core keywords**	**Number of papers in the database**	**Papers matching the research topic**	**Literature that is highly relevant to the research topic**
Top 1: online deviance OR cyber deviance OR online deviant behavior	250	25	4
**Combined keywords**	**Number of papers in the database**	**Papers matching the research topic**	**Literature that is highly relevant to the research topic**
Topic 1: cyber deviance AND digital behavior AND moral	0	0	0
Topic 2: online deviance AND digital behavior AND moral	1	1	0
Topic 3: online deviance OR cyber deviance, digital behavior AND moral	1 (the same article as topic 2)	1 (the same article as topic 2)	0
Topic 4: online deviant behavior AND moral	12	3	0
**Related keywords**	**Number of papers in the database**	**Papers matching the research topic**	**Literature that is highly relevant to the research topic**
Internet plagiarism OR Online plagiarism	104	19	5
Internet trolling OR Online trolling	229	52	2
Cyberbullying	937	286	26
Online harassment OR Internet harassment	796	65	38
Academic misconduct	263	40	8
Online cheating	182	34	4
Flaming	461	12	1

Notably, considering the emerging and diverse nature of the topics involved in our research, most of the highly cited literature included in the final sample from Google Scholar had cumulative citation counts exceeding 20. This aligns with our research objective of using Google Scholar to retrieve representative literature involving relevant keywords, where the primary reference criterion for literature included in the final sample after Google Scholar retrieval was the literature's compatibility with the research objectives, while citation count served as a secondary reference criterion.

More articles from Google Scholar were included in the final sample. However, this does not reflect selection bias, as our goal was not to balance the number of articles by database but rather to identify representative studies that can inform the conceptual model of online moral deviance. As Snyder (2019) notes, integrative reviews are not expected to exhaustively cover all relevant literature but rather to synthesize insights across disciplines through a strategic and purposive selection of studies.

To reduce subjectivity in the screening process, we developed a detailed screening manual. Two reviewers independently evaluated the titles and abstracts of all retrieved articles against predefined evaluation criteria. Any discrepancies between the reviewers were discussed thoroughly until consensus was reached.

## 3 Research findings

A review of existing literature indicates that, to date, the term online moral deviance has not been explicitly proposed, nor does it have a unified definition. In our study, *online moral deviance* refers to a range of digital behaviors that violate the moral expectations widely accepted within the mainstream culture of a specific society. Based on literature search and analysis, the digital behaviors constituting online moral deviance primarily include internet plagiarism, internet trolling, cyberbullying, online harassment, academic misconduct, online cheating, flaming, and so forth. These digital behaviors are characterized by unpredictability, intentionality, and harmfulness, all of which negatively impact online target users. Their specific manifestations have been identified in previous research. Identifying and integrating these digital behaviors, as well as constructing a conceptual model for them, contribute to a deeper understanding of and a more effective response to this complex digital behavior pattern.

Based on the moral framework, our research identified, categorized, and elucidated the interconnected logical relationships among digital behaviors involved in cyber deviance, online deviance, and online deviant behavior across three dimensions: the moral characteristics exhibited by digital behaviors, the primary manifestations of violations against moral expectations commonly recognized in mainstream culture within specific societies, and the main harm caused to targeted online users. The categorization of digital behaviors under the research themes of cyber deviance, online deviance, and online deviant behavior based on these three dimensions provides explanatory grounds for why certain digital behaviors can constitute components of online moral deviance and why certain digital behaviors can be used to construct the conceptual framework of online moral deviance.

### 3.1 Components of online moral deviance

Choi and Park (2023) concluded in their study that individuals with different characteristics exhibit significant differences in the average levels of Internet ethics. This suggests that individuals may exhibit varying forms and degrees of unethical deviant behavior online, contingent upon their distinct levels of internet ethics. In our research, online moral deviance is conceptualized as a comprehensive pattern of digital deviant behaviors. These behaviors encompass, but are not limited to, key elements such as Internet plagiarism, Internet trolling, cyberbullying, online harassment, academic misconduct, online cheating, and flaming. Each of these elements represents a unique form of deviant behavior within the digital space. After outlining the components of online moral deviance, our research will also develop a conceptual model to facilitate a clearer understanding of this integrated pattern of digital behavior.

#### 3.1.1 Internet plagiarism

Prior research has thoroughly investigated the traditional definitions, specific behaviors, and underlying causes of plagiarism. Some studies are particularly representative. Roig (2012) emphasized that plagiarism refers to the dishonest use of another individual's intellectual property without authorization. It encompasses original works such as texts, charts, graphics, research methodologies, theoretical concepts, and other intellectual contributions. According to Howard (1999, p. 25), plagiarism may take the form of “patchwriting,” which involves rewriting and paraphrasing, where source texts are reproduced with modifications to their content and structure. Results of previous studies have shown that common causes of plagiarism include lack of time (Memon and Mavrinac, 2020), lack of methodology, lack of awareness, neglect of citation requirements, insufficient specialized training, absence or ineffective enforcement of penalties, anxiety about failure, low self-confidence, laziness, a norm of academic dishonesty (Malik et al., 2021), lack of search skills, pressure from publication requirements, pressure from assessment (Osuchukwu et al., 2022), and the tendency to use online platform materials unethically (Sorea and Repanovici, 2020). However, this behavior is generally viewed as immoral regardless of who commits it (Shahabuddin, 2009).

Accompanied by the convenience of accessing online resources, the act of plagiarism has extended into the realms of online learning and research, a digital behavior has arisen—Internet plagiarism. It is a form of dishonest digital behavior that relies on emerging technologies and digital devices. According to Scanlon and Neumann (2002), internet plagiarism refers to the act of copy and paste text from the internet into academic papers without proper citation, as well as purchase papers online. Sisti (2007) contend that internet plagiarism involves using the internet to copy assignments or purchase completed work. Internet plagiarism encompasses various practices, include copying digital materials, paraphrasing original texts, translating original texts into other languages, trancoping or paracopying original texts, and combining or splitting digital materials without acknowledging the original authors (Eret and Ok, 2014). Additionally, also includes fabricating information. Relevant research findings also indicate a close connection between online plagiarism and offline plagiarism as self-reported by individuals (Selwyn, 2008).

Based on previous literature findings, Internet plagiarism exhibits characteristics of dishonesty, unpredictability, and intentionality. It is evident that Internet plagiarism is an unethical digital behavior involving the use of the Internet to copy, modify, or purchase others' works or ideas after copying or purchasing, and then presenting them as one's own achievements. The characteristics and specific manifestations of Internet plagiarism indicate that it violates the widely accepted moral expectation of honesty within the academic and intellectual property domains, as upheld by the dominant culture of a given society.

#### 3.1.2 Internet trolling

At present, scholars have not reached an agreement on the definition of trolling (Ortiz, 2020). Some researchers suggest that “trolling” refers to a collection of hostile, antisocial, and deviant behaviors and motivations (e.g., Buckels et al., 2014; Phillips, 2015; Sanfilippo et al., 2018; Paakki et al., 2021). However, there are some scholars who believe that the purpose of trolling may be to provoke conflict, or it may be to elicit interactions that the pranksters find amusing. For instance, Coles and West (2016) argue that current trolling is perceived as “nasty,” whereas in the past, it was regarded as elegant. Therefore, some scholars argue that trolling behavior is context-dependent (Hardaker, 2010). In this regard, some scholars suggest that trolling should be defined based on user discussions (e.g., Culpeper, 2011; Hardaker, 2010).

Currently, the prevailing view among researchers is the definition of trolling should primarily be based on the information provided by users or respondents. Ortiz (2020) claims that surveys reveal that respondents generally believe trolling is not an attempt to joke with targets in a humorous manner, nor merely an effort to divert conversations from general matters, but rather a form of collective harassment. Trolls are perceived as instruments of provocation, employed by perpetrators to derail targets from meaningful public discourse, with the intention of inducing anger, frustration, or silence, thereby causing harm to the targets (Ortiz, 2020). The most commonly described feelings words of trolling victims are feel, annoyed, effect, upset, and angry (March and Marrington, 2019). Trolling is widely perceived as an insulting, bullying, and deliberate behavior (March and Marrington, 2019). Intentionality and deception are defining characteristics of trolling (Golf-Papez and Veer, 2017). This reflects the feelings and understanding of trolling among respondents in some empirical studies. Research trends indicate that trolling has evolved from mutual enjoyment or entertainment-oriented act to a form of aggressive abusive behavior (Bishop, 2014).

Internet trolling, also known as online trolling, or simply trolling, occurs in online social environments. Relevant research findings are primarily concentrated in the discipline of psychology. Through word frequency analysis, March and Marrington (2019) confirmed that Internet trolling is an aggressive behavior characterized by abusiveness. Previous research has demonstrated that trolling encompasses a wide spectrum of behaviors. Thacker and Griffiths (2012) found that this type of behavior encompasses irritating behavior, gender discrimination, intentional falsification, and misleading claims. Ortiz (2020) emphasize that this type of behavior also include hacking, disclosing personal details, posting sarcastic remarks, submitting redundant material, and hate speech.

Based on the literature review, the dominant perspective is that the aim of internet trolling is to stir up conflict and provoke anger in the target. Internet trolling may be considered both unpleasant and unethical (Coles and West, 2016). It has a profound adverse impact on the mental health of the individuals targeted (Wu et al., 2022). The results of previous studies have revealed the nature of Internet trolling to be unethical and harmful. Building on previous research, internet trolling can be considered a form of intentional, unethical, negative and deviant digital behavior in online social environments—such behavior has a provocative character that negatively affects the perceiver. It aims to stir up conflict, provoke anger and even harm the target. It should be noted that this depends on the perpetrator's intentions, the context in which the term is used, and the reactions of users or respondents. Based on the existing definitions, characteristics, purposes of Internet trolling, and the harm it causes to online target users, it violates the moral expectations of respect and empathy.

#### 3.1.3 Cyberbullying

Smith et al. (2008) define cyberbullying as a type of deliberate, long-term, and repeated aggressive behavior inflicted by individuals or groups through digital means on victims who are unable to fully protect themselves. This definition is representative, it is widely regarded as an adaptation of the definition originally proposed by Dan (1993) for traditional bullying. It is characterized by intentionality and repetition (e.g., Erdur-Baker, 2010), compared to traditional bullying, it also highlights characteristics such as anonymity, spatiotemporal flexibility, and high transmissibility (Dou et al., 2020). Its purpose is to cause harm or discomfort on targeted Internet users (Kowalski et al., 2008; Hinduja and Patchin, 2012; Olweus and Limber, 2018; Huang et al., 2019; Dou et al., 2020; Wang and Ngai, 2021; Chan et al., 2021). Compared to traditional bullying, cyberbullying poses a more severe threat to targeted Internet users due to its lack of spatiotemporal constraints. The harmful consequences of experiencing cyberbullying include various psychological and behavioral outcomes. For instance, the research by Farrington et al. (2023), revealed that notable impacts encompass anxiety, depression, drug use, suicidal tendencies, and academic underperformance. Similarly, Celuch et al. (2024) highlighted stress, technical pressure, work-related burnout, and decreased work motivation as key consequences.

Perspectives based on social psychology, in the case of bullying and cyberbullying, conflict is at the root of that behavior, and it involves individuals who have chosen an aggressive strategy to deal with it (Summers, 2017). Sureda Garcia et al. (2020), for example, emphasize that interpersonal conflicts stemming from excessive internet use may result in cyberbullying. Recent psychological findings suggest that cyberbullying is associated with strain and levels of self-control (Li and Peng, 2022), low self-control (Cho and Glassner, 2021), social status among peers (Wang and Ngai, 2021), paranoid tendency (Lin and Xiao, 2023), childhood emotional abuse and Problematic Social Media Use (Xu and Zheng, 2022), trait anger (Yang et al., 2022a), encounter stressful situations in life (Geng and Lei, 2021), violent video game exposure (Teng et al., 2020), problematic Internet use (Yudes et al., 2022), childhood cyberbullying victimization (Lee, 2021), having a conflict with parents (Xue et al., 2022), materialism and parental psychological control (Geng et al., 2022). It is also positively associated with workplace bullying, role confusion, daily stress, and turnover intention (Czakert et al., 2021).

Some researchers have also argued that cyberbullying is often driven by moral anomie. Internet users often use “justice” as a standard for moral judgment, but if moral judgment is irrational, it may cause cyberbullying (Zhan et al., 2022). Cyberbullying manifests in various forms. The main forms of cyberbullying include transmitting malicious digital text content, disseminating offensive visual materials, spreading online rumors, online social exclusion, unauthorized disclosure of personal information online, and online identity deception (Patchin and Hinduja, 2010; Perren et al., 2012; Zych et al., 2020), online harassment (Olweus and Limber, 2018), flaming, outing, miss information and cyberstalking (Newey and Magson, 2010). In addition, online trolling or Internet trolling is also viewed a form of cyberbullying (e.g., Morissey, 2010; Griffiths, 2014; March and Marrington, 2019).

Past studies have consistently held that ethical factors are interrelated with cyberbullying. Current studies have established that moral disengagement as a crucial factor in predicting cyberbullying conduct (Luo and Bussey, 2019; Bussey et al., 2020; Cricenti et al., 2022). Lo Cricchio et al. (2020), for example, underscored that among adolescents, the disposition toward moral disengagement represents the most salient personality trait that contributes to youth engage in cyberbullying. According to Llorent et al. (2021), weak moral emotions are a risk factor for committing cyberbullying. Zhang et al. (2020) validated that positive moral personality traits were significantly inversely correlated with attitudes toward cyberbullying perpetration. Recent findings suggest that the moral disengagement was positive associated with cyberbullying (e.g., Paciello et al., 2020; Wang and Ngai, 2020; Gao et al., 2020; Lo Cricchio et al., 2020; Lin and Xiao, 2023; Zhao and Yu, 2021). Recent studies have also shown that ethical factors can influence cyberbullying between different roles. Li (2023), for instance, mentioned that moral disengagement could be a key factor influencing the shift from being a victim of cyberbullying to becoming a perpetrator. Moreover, moral disengagement also serves as a mediator in the relationship between specific factors and cyberbullying (Liang et al., 2022), such as paranoid tendencies (Lin and Xiao, 2023) and peer pressure (Yang et al., 2022b). Previous literature also suggests that the motivation behind individual deviant behavior may be associated with personal moral inclinations (Luo et al., 2023).

Cyberbullying is one type of deviant behaviors and its intentions may be affected by moral factors (Luo et al., 2023). The literature review indicates that cyberbullying is a form of malicious digital behavior that is intentional, repeated, and prolonged, targeting victims who are unable to defend themselves through electronic means. It is associated with moral factors and possesses hostile or hurtful attributes. Based on existing research findings on the definition, manifestations, and harm to online target users of cyberbullying, cyberbullying violates the moral expectations of kindness, empathy, and respect.

#### 3.1.4 Online harassment

Previous literature has provided the following representative explanations of online harassment, mainly from a social psychological perspective. Summers (2017) argue that online harassment is a form of conflict behavior driven by incompatible or opposing interests, goals, or needs. Conflicts in the online environment are one of the causes of online harassment (Lee et al., 2022). Nadim and Fladmoe (2021) contended that online harassment is an overarching behavioral concept that encompasses a range of negative online behaviors, including mild negative comments, severe hate speech, and threats. The core characteristics of online harassment are anonymity, long-term effects, and widespread negative impacts (Gagliardone et al., 2015; Cricenti et al., 2022).

The main victims of online harassment are young people and women. Typically, men are more likely to perpetrate online harassment, while women experience it more severely (van Baak et al., 2023). Posited that name-calling and physical threats are the main forms of cyberbullying experienced by men, while women are primarily affected by sexual harassment. Lee et al. (2022) illustrated that significant predictors of online harassment include premeditated aggression, impulsivity, impulsive aggression, and age.

Furthermore, according to Nadim and Fladmoe's (2021) framework, online harassment can be categorized based on two key aspects. The first one is the level of aggressiveness in the tone or style of the comment. The second one is what the comment targets. The first dimension reveals the aggressiveness of online harassment. It encompasses a range of comments, from mild remarks that make the recipient feel uncomfortable to more severe and malicious hate speech. The second dimension pertains to the content of the comments, including but not limited to the target user's opinions, appearance, and ideological stance.

Different targets of online harassment have been distinguished by some scholars. The targets of group-based online harassment encompass: age, race, gender, color, religion, ethnicity, nationality, disability, or sexual orientation. The targets of individual-based online harassment include: individual's personality and/or individual's appearance, attitudes or arguments (Nadim and Fladmoe, 2021). Recently, group-based online harassment has seen scholars focus their research primarily on these groups: journalist (e.g., Lewis et al., 2020; Kantola and Harju, 2023; Holton et al., 2023; Sammut et al., 2023; Li et al., 2023; Uwalaka and Amadi, 2023; Uwalaka et al., 2023; Bhat, 2024; Dodds et al., 2024; Shah et al., 2024; Lee and Park, 2024; Yeon Lee and Park, 2024), especially female journalists (e.g., Koirala, 2020; Chen et al., 2020; Miller and Lewis, 2022; Siddiqua et al., 2023; Tandoc et al., 2023; Zviyita and Mare, 2024; Sampaio-Dias et al., 2024), media professionals (e.g., Celuch et al., 2023), adolescents (e.g., Vale et al., 2022; Choi et al., 2022; Pazhouhi, 2023; Waechter and Meschik, 2023), academics (e.g., Gosse et al., 2021; Houlden et al., 2022; Oksanen et al., 2022; Eslen-Ziya et al., 2024), feminist academics (e.g., Dej and Kilty, 2024), women of color (e.g., Francisco and Felmlee, 2022), politicians (e.g., Wagner, 2022), bystander (e.g., Brody, 2021; Spaccatini et al., 2023), audience member (e.g., Lu and Luqiu, 2023) and famous people (e.g., Takano et al., 2024).

Online harassment, which aims to annoy, abuse, and torment individuals in cyberspace, can lead to significant negative consequences. The potential consequences, particularly psychological ones, include victims becoming unwilling to participate in public discussions and express their views (Nadim and Fladmoe, 2021), depression, anxiety, suicidal thoughts, panic attacks (Stevens et al., 2021), psychological distress, low trust, and decreased perception of social support (Oksanen et al., 2022). Prior research findings also indicated that the consequences of group harassment are broader and more damaging (Nadim and Fladmoe, 2021), with the perceived harm from repeated harassment being more pronounced (Schoenebeck et al., 2023).

A literature review indicates that online harassment lacks a universally accepted definition. However, from the proposed definitions, some predictors of online harassment and negative impact of being subjected to cyber harassment, there is evidence to suggest that it is an unethical digital behavior within a moral framework. Based on research findings on the behavioral manifestations of online harassment, online harassment violates the moral expectations of empathy, respect for individual rights, respect for personal privacy, and non-discrimination.

#### 3.1.5 Academic misconduct

It was discovered that there is no consensus definition of academic misconduct after a review of previous research. Stephens et al. (2021), for example, proposed that academic misconduct refers to engaging in behaviors such as plagiarism and cheating, which are explicitly prohibited by institutions. Krou et al. (2021) mentioned that academic misconduct is a motivated behavior that is deeply influenced by a sense of purpose. The common academic misconduct includes copying, cheating, plagiarism, collaboration, distorted information (Ramim, 2005), employing unauthorized materials, acquiring exam questions or answers through unethical methods prior to the examination (Keresztury and Cser, 2013), illicit collusion, impersonation, fabrication or falsification (Siddhpura and Siddhpura, 2020), communicating with external helpers during an exam (Keresztury and Cser, 2013), fake acceptance letter and financial fraud (Stockemer and Reidy, 2024).

Academic misconduct related to digital technology can take many forms or operational behaviors, it includes unauthorized using of digital resources, cheating or plagiarizing using digital technology, online collusion, and online deception using someone else's username and password (Ramim, 2005), misuse of portable storage devices, viewing answers displayed on different computer monitors (Keresztury and Cser, 2013), screen sharing (Siddhpura and Siddhpura, 2020), improper citation practices, tampering with text-matching software (Luck et al., 2022), Internet plagiarism (e.g., Huang et al., 2024), duplicate publication, self-plagiarism, self-citation, quote manipulation (Feenstra et al., 2021), Internet-based assignment outsourcing (Awdry, 2021).

Contract cheating is considered one of the most challenging issues faced by academic professionals (Amigud, 2020). It is also a widely studied form of academic misconduct. Clarke and Lancaster (2006) defined contract cheating as the act of students paying contractors to complete assignments on their behalf in exchange for academic credit. Ison (2020) defined contract cheating as students hiring external individuals to complete their academic assignments. Walker and Townley (2012) noted that contract cheating refers to the act of students outsourcing their coursework to writers and submitting the purchased work as their own through the internet, which raises ethical concerns. Due to advancements in end-to-end encryption technology in digital communication, contract cheating has become increasingly prevalent and more difficult to detect (Siddhpura and Siddhpura, 2020). Contract cheating is evolving and spreading in the context of the internet. It is clear that contract cheating, within the internet environment, constitutes a significant component of online academic misconduct.

The rising number of academic misconduct cases not only negatively impacts academic integrity but is also becoming a moral issue worthy of attention (Siddhpura and Siddhpura, 2020). Academic misconduct is a concrete manifestation of negative morality (Stephens and Bertram Gallant, 2024). Zhang et al. (2024), for example, concluded that individuals who have experienced moral disengagement are more likely to exhibit plagiarism using AI tools. It can be seen that academic misconduct is a notable digital behavior that goes against ethical norms. The specific manifestations of academic misconduct indicate that it violates the moral expectations of honesty, responsibility, and trustworthiness.

#### 3.1.6 Online cheating

It is known from previous studies that cheating is an unethical behavior. Srikanth and Asmatulu (2014), for example, posited that cheating is an unethical behavior that violates rules and regulations and aims to gain an advantage in competition by unfair means. Traditional cheating is manifested as copying information from paper, using disabled notes in exams, copying someone else's answers (McCabe, 1999), Cheating behavior is usually found in homework tasks, exams, essay writing, project reports, presentations (Srikanth and Asmatulu, 2014). The progress of internet technology has enabled the widespread use of online learning and research. Traditional offline cheating has transitioned to the online setting, manifesting in new and more complex behaviors. This has given rise to a form of unethical digital behavior—online cheating. Online cheating also known as cyber-cheating or digital cheating.

Copying online electronic resources, plagiarizing others' online work, buying essays or homework answers online (Young, 2001), using remote desktop, searching for solutions using the Internet (Noorbehbahani et al., 2022), credential sharing (Dobrovska, 2017), using problem solving apps or websites for plagiarism, sending and having others assist in completing test questions or tasks (Bawarith et al., 2017), and using offline electronic resources (Holden et al., 2021) are all ways of digital cheating. According to Srikanth and Asmatulu (2014), the tools used for digital cheating arephones, earphones, MP3 players, graphing calculators, iPads, texting devices multifunctional watches and other digital communication devices. According to Zayed (2023), digital collective cheating is also a category of digital cheating which deals with the ethics of cooperation. Noorbehbahani et al. (2022) conducted a systematic review focused on online cheating, evaluating and synthesizing relevant literature from 2010 to 2021. Their review offers comprehensive insights into cheating motivations, types, detection methods, and preventive measures within online educational environments.

Contract cheating has been recognized in academia as a significant form of online cheating, for it has distinct online transaction characteristic. According to Clarke and Lancaster (2006), contract cheating is the act of an individual outsourcing coursework, papers, or other tasks that need to be completed to a third party via the Internet. The method of contract cheating is outsourcing assignments. According to Awdry (2021), contract cheating is done through essay mills, custom assignment services, essay bidding services, and peer sharing sites.

Previous literature has analyzed the impact factors or motivations for individual or collective involvement in cheating. According to Wang and Xu (2021), expectations regarding academic performance, satisfaction with assignments, and anticipated learning outcomes are all factors that influence whether students engage in cheating behaviors. Research findings by Yazici et al. (2023) demonstrated a significant relationship between gender, academic discipline, and cheating behaviors. The study conducted by Navidinia et al. (2024) revealed that stress and time constraints were the primary motivations for cheating.

According to a review of existing literature, the types of online cheating are diverse. Such behavior negatively impacts the authentic and fair evaluation of students' academic achievements and researchers' scholarly performance. It is a deceptive digital behavior rooted in the convenience of accessing online resources and their unethical use, violating ethical standards. The definition and operational behaviors of online cheating demonstrate that it violates the moral values of fairness and integrity.

#### 3.1.7 Flaming

The meaning of Flaming has been well explained in previous literature, and some scholars' explanations are representative. According to Kayany (1998), flame is an uninhibited expression of hostility. Petit et al. (2021) proposed that flaming refers to a strong and hostile expression of emotions and feelings online. Bell et al. (2004) stated that flaming is a variety of messages in cyberspace that use abusive language. Based on those concepts given by previous scholars, Ardi et al. (2020) posited that flaming can be concluded as an utterance that expresses insults, swear words, and hateful, intense language in hostile online interaction. Flaming is described as a highly negative message (O'Sullivan and Flanagin, 2003). Hostile expression of strong emotions and feelings are the conditions under which flaming occurs (Lea et al., 1992). The primary behaviors of flaming operations include swearing, calling names, mocking or insulting others' appearance, religious beliefs, intellectual level, ethnicity, and physical or mental abilities (Kayany, 1998).

In the discussion of the definition of flaming, Thompsen (1993) stated that a 'true flame' refers to a message intentionally sent by the sender that violates interaction norms, and is perceived by the receiver as a transgression of these norms. Some scholars such as O'Sullivan and Flanagin (2003) support this view, emphasized that in determining whether behavior is flaming, we need to consider both the sender's intent and the receiver's perception. By combing through the literature on the conceptualization of flaming, Andersen (2021) affirmed that early definitions of flaming were typically based on textual elements, but later approaches emphasized the speaker's intent and the target user's experience as the core components of the definition. Based on previous discussions of the concept of flaming in the literature, flaming is intentional, norm-violating, and unrestrained in nature, and emphasizes the intent of the perpetrator and the experience of the perceiver of the act.

Flaming has different characteristics, and linguistic approaches can be combined when it comes to recognizing or determining whether a behavior is associated with flaming. Andersen (2021) suggested that combining features of the text and the context of the text to test whether flaming is involved. Zhang (2021) advocated that implicit invisible flaming can be recognized in terms of keywords, semantic prosodies and speech acts. From a linguistic point of view, most studies claim that the features of flaming mainly contain “hostility, aggression, intimidation, insults, offensiveness, unfriendly tone, uninhibited language, and sarcasm” (Turnage, 2007, p. 44). Linguistic approach can help in recognizing flaming.

The category of Flaming has been divided by some scholars in their studies. Depending on the nature of the act and the manner, flaming is generally categorized as direct and intentional flaming and indirect flaming (e.g., Bansal et al., 2012; Ardi et al., 2020). Based on the findings of Lingam and Aripin (2017), and taking into account operational behavior, Ardi et al. (2020) categorizes flaming into 14 subcategories: “stereotype; speculation; comparison; degrading; defamation; sedition; sarcasm; threaten; challenge; criticism; name-calling; sexual harassment; satirical; and straightforwardness” (p. 120). The main mediums of Flaming include email, news sites, Twitter, message boards and YouTube (Petit et al., 2021). Through empirical studies, several researchers have found that some of the motivations that contribute to flaming includes: “excessive fanaticism, self-interest, spontaneous emotions and anxiety, low digital literacy, the anonymity of netizens on social media through second accounts, and lack of empathy” (Arisanty and Wiradharma, 2022, p. 215), personality traits, pass time, escape, relaxation and entertainment (Alonzo and Aiken, 2004).

Prior research has shown that Flaming has a variety of manifestations and a wide range of operational behavior. It encompasses behaviors ranging from minor breaches of norms, such as impolite comments, to more severe forms of insults, such as abusive remarks (Papacharissi, 2004). Its operational behavior mainly includes sending messages indiscriminately that are characterized by hostility, aggression, intimidation, and insults (Turnage, 2007), uncivil or offensive comments and discussions in online conversations or online discussions (Petit et al., 2021), using offensive language, making vulgar jokes, and delivering sarcastic remarks (Leite et al., 2023).

Previous literature suggests that flaming violates the moral norms of mainstream society, and it is a form of cyber deviance. For instance, the results of a study on cyber deviance among adolescents conducted by Chen et al. (2021) showed that online flaming is prevalent among adolescents and constitutes a form of cyber deviance. Grounded in linguistics, Ardi et al. (2020) conducted a study focused on the speech act of flaming, analyzing this phenomenon within Twitter statuses in the Malaysian context. The research findings indicate that the language used by the adolescent group of Twitter users does not align with the values and moral standards promoted by culture and society.

By considering the definitions, characteristics, categories, and forms of flaming, it becomes clear that these speech acts, which deviates from the moral standards recognized by mainstream culture, represent a form of moral deviance. This digital behavior violates the moral values of politeness, respect for others, and rational communication. Therefore, we categorize flaming as a digital behavior that falls within the realm of online moral deviance, viewing it as a specific type of online moral deviance.

Table 2 below provides a more intuitive overview of the core aspects of each component within the concept of online moral deviance. These aspects illustrate the rationale for categorizing these behavioral types as integral elements of online moral deviance.

**Table 2:** The core content framework of each component of online moral deviance.

**Components of online moral deviance**	**Some typical manifestations of violating mainstream cultural moral norms**	**Characteristic**	**Primary harm caused to the target user**
Internet plagiarism	Unattributed online copy-paste, purchase papers online (Scanlon and Neumann, 2002), copy assignments online or purchase completed work online (Sisti, 2007), and breaking down digital materials (Eret and Ok, 2014).	Unpredictability, intentionality, and dishonesty (Roig, 2012).	It can infringe on the intellectual property rights of the original author's digital work.
Internet trolling	Gender discrimination, misleading claims (Thacker and Griffiths, 2012), hacking, disclosing personal details, posting sarcastic remarks, submitting redundant material, and hate speech (Ortiz, 2020).	Antagonism (Buckels et al., 2014), intentionality and deception (Golf-Papez and Veer, 2017).	①Cause the target users to experience anger, frustration, or silence (Ortiz, 2020). ②Cause severe negative impacts on the target individual's mental health (Wu et al., 2022).
Cyberbullying	Transmitting malicious digital text content, disseminating offensive visual materials, spreading online rumors, online social exclusion, unauthorized disclosure of personal information online, and online identity deception (Patchin and Hinduja, 2010; Perren et al., 2012; Zych et al., 2020).	Intentionality, repetition (Erdur-Baker, 2010), anonymity, spatiotemporal flexibility, and high transmissibility (Dou et al., 2020).	Notable impacts encompass anxiety, depression, drug use, suicidal tendencies, and academic underperformance (Farrington et al., 2023); distress, technical pressure, work-related burnout, and decreased work motivation (Celuch et al., 2024).
Online harassment	Mild negative comments, severe hate speech, and threats (Nadim and Fladmoe, 2021). Name-calling, physical threats and sexual harassment (Powell and Henry, 2015).	Anonymity, long-term effects, and widespread negative impacts (Gagliardone et al., 2015; Cricenti et al., 2022).	Depression, anxiety, suicidal thoughts, and panic attacks (Stevens et al., 2021). psychological distress, low trust, and decreased perception of social support (Oksanen et al., 2022).
Academic misconduct	Duplicate publication, self-plagiarism, self-citation, quote manipulation (Feenstra et al., 2021), Internet-based assignment outsourcing (Awdry, 2021).	Prevalence, pervasiveness, concealment (Siddhpura and Siddhpura, 2020) and deceptiveness.	Negatively impacts academic integrity (Siddhpura and Siddhpura, 2020).
Online cheating	Copying online electronic resources, plagiarizing others' online work, buying essays or homework answers online (Young, 2001), using remote desktop (Noorbehbahani et al., 2022).	Online transaction, concealment and deceptiveness.	It undermines personal integrity, diminishes independent thinking and problem-solving abilities, and may negatively impact mental health. It disrupts a fair competitive environment.
Flaming	Swearing, calling names, mocking or insulting others' appearance, religious beliefs, intellectual level, ethnicity, and physical or mental abilities (Kayany, 1998).	Offensiveness (Turnage, 2007), intentionality, norm violation and lack of constraints.	Uncivil or offensive comments and discussions in online conversations or online discussions (Petit et al., 2021).

Table 2 illustrates that the behaviors constituting the core components of online moral deviance exhibit typical manifestations of violating the moral norms recognized by mainstream culture. These behaviors are not accepted or endorsed by mainstream cultural values. The key components of online moral deviance are characterized by distinct negative attributes, including intentionality, unpredictability, repetitiveness, deception, and aggression. Such behaviors can inflict varying degrees of physical, emotional, or psychological harm on online targets. Therefore, these behaviors are identified as fundamental elements of online moral deviance.

### 3.2 Conceptual model

Deviance refers to behavior that deviates from social norms, which can lead to public disapproval and potentially result in negative consequences or punishment (Clinard and Meier, 2011). According to Plé and Demangeot (2020), deviant behavior can be viewed as any behavior by an individual or a member of an organization that is perceived as violating social norms, organizational policies, or laws, or disrupting their own or others' functional experiences, regardless of intent, falls within this scope.

The deviance discussed in our study specifically focuses on the moral domain, and these deviant behaviors occur online. Based on Parsons' (1951) research on the characteristics of deviance, the term deviance in the present study refers to behaviors occurring in online environments that violate the moral norms of mainstream culture. The different types of deviant behaviors included in our research are supported by a substantial research foundation, as they have been widely addressed in fields such as education, psychology, sociology, and management, providing a scientific basis for the present study.

Building on the definitions of deviance and deviant behavior outlined in the existing literature, the present study categorizes internet plagiarism, internet trolling, cyberbullying, online harassment, academic misconduct, online cheating, and flaming as different types or components of online moral deviance. This categorization is based on the following criteria: (1) these behaviors diverge from the moral and social norms of mainstream culture, significantly reflecting the characteristics of deviance. (2) these behaviors violate widely accepted moral standards or socially normative expectations within an online community. (3) whether intentional or unintentional, these behaviors possess negative attributes such as conflict, harm, and aggression, which can negatively impact individuals and others or disrupt functional experiences (Plé and Demangeot, 2020). These criteria are grounded in representative definitions of deviance and deviant behavior from previous literature.

**Figure 2:**
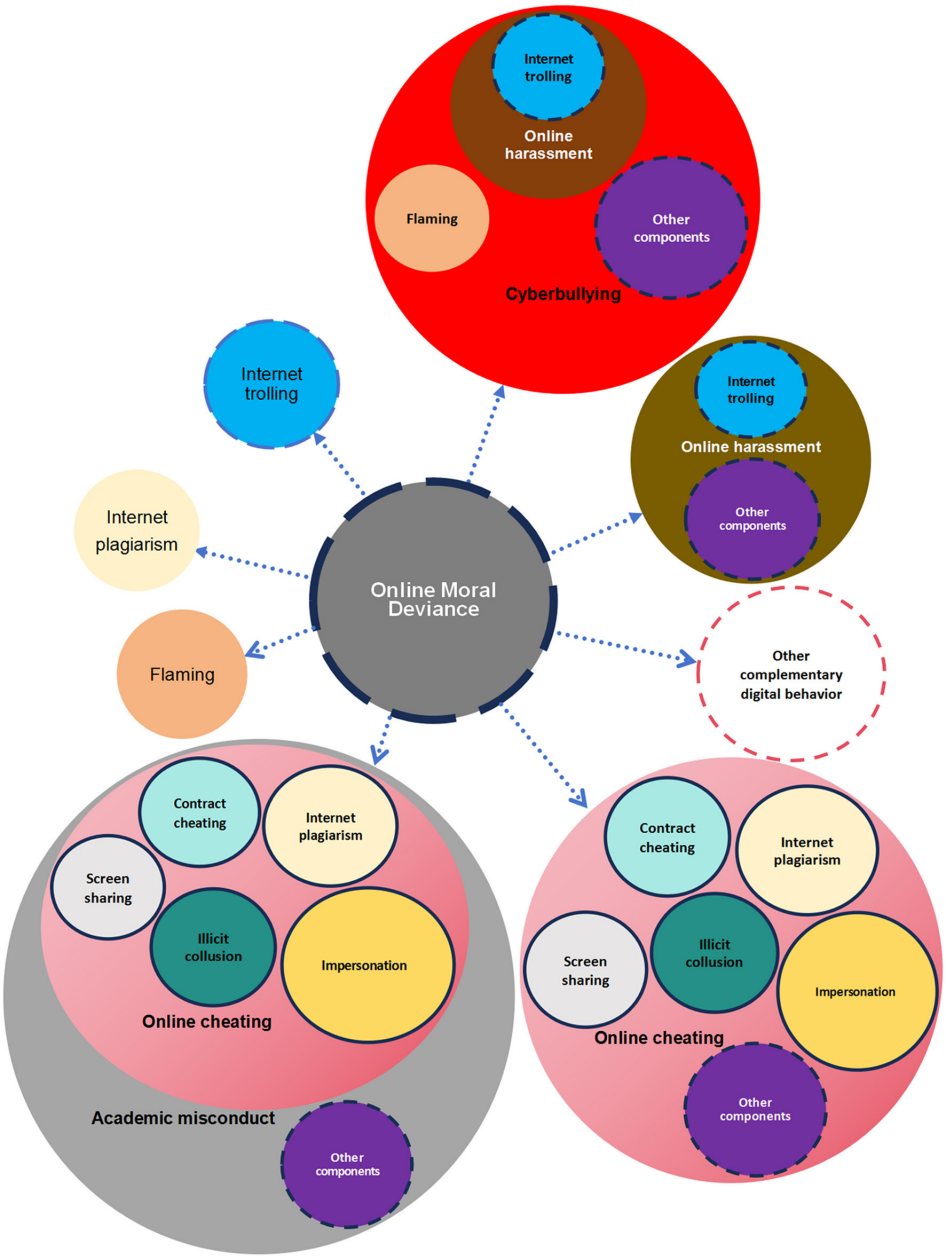
The conceptual model of online moral deviance.

To understand the multifaceted nature of online moral deviance, our study proposes a conceptual model that encompasses several key elements: internet plagiarism, Internet trolling, cyberbullying, online harassment, academic misconduct, online cheating, and flaming. However, this model is not limited to these specific components. It not only describes these elements but also emphasizes their interconnections, indicating that individuals may engage in multiple forms of online moral deviance. Understanding these components and their relationships is crucial for developing effective interventions aimed at mitigating online deviant behaviors and fostering a healthier digital environment. Figure 2 below is a conceptual model of online moral deviance constructed from a review of previous literature.

In the conceptual model of online moral deviance is open to integrative expansion and comprises not only elements such as internet plagiarism, trolling, cyberbullying, online harassment, academic misconduct, online cheating, flaming, and other criteria-based digital behaviors mentioned above can be added. Within this model, the various elements—or components—exhibit relationships of inclusion or coexistence, where one element may encompass multiple others. For example, some scholars have consistently agreed that elements constituting online cheating also include screen sharing (e.g., Noorbehbahani et al., 2022), illicit collusion (e.g., Siddhpura and Siddhpura, 2020), impersonation (e.g., Dobrovska, 2017), and other supplementary components. Prior studies (e.g., Nwosu and Chukwuere, 2020; Stephens et al., 2007; Clarke and Lancaster, 2006) have also identified online plagiarism and contract cheating as distinct forms of online cheating. Previous studies have also shown that academic misconduct encompasses online cheating (e.g., Siddhpura and Siddhpura, 2020). Additionally, a growing body of research has consistently identified internet trolling can be viewed as a type of online harassment (e.g., Gray, 2012; Gray et al., 2017; Vera-Gray, 2017; Manuoglu and Öner-Özkan, 2022), or a specific subset of online harassment (e.g., Ortiz, 2020; Manuoglu and Öner-Özkan, 2022). Some studies also highlight that Internet trolling may also be categorized as a type of cyberbullying (e.g., Morissey, 2010; Griffiths, 2014). Several studies have consistently recognized that flaming (e.g., Johnson et al., 2008; Zhang, 2021) and online harassment (e.g., Newey and Magson, 2010) are likewise forms of cyberbullying. Clearly, subsets of cyberbullying also include other integrative components.

In the conceptual model of online moral deviance, the primary categories can expand further, allowing for the inclusion of additional digital behaviors that meet the criteria for deviance. This indicates that the model of online moral deviance is not static; rather, it evolves in response to the changing landscape of digital behaviors that align with the criteria for deviance.

## 4 Discussion

Existing literature on cyber deviance or online deviance has primarily focused on identifying the factors that influence participation in online deviant behaviors (e.g., Holt et al., 2012; Lee, 2018; Chen et al., 2021; Virgara and Whitten, 2023; Whitten et al., 2024) and the relationship between cyber deviance and these influencing factors (e.g., Holt et al., 2012; Chen et al., 2021; Louderback and Antonaccio, 2021; Whitten et al., 2024). As Cioban et al. (2021) noted that social scientists often utilize classical theories of deviance to analyze this phenomenon, focusing on identifying key predictors and examining their interactions across both online and offline environments.

However, when it comes to online deviance or cyber deviance, most systematic reviews focus on specific types of behaviors (Cioban et al., 2021). Indeed, deviant behaviors range from serious crimes classified as delinquent to minor antisocial behaviors that are not punishable by the criminal justice system (Gorman-Smith et al., 1998). While a substantial amount of research has been conducted on deviance, the focus of existing research is often narrow. It is necessary to provide a more structured and systematic introduction to the common themes in this field (Cioban et al., 2021). A key gap in prior research is the lack of a comprehensive framework that considers these morally deviant digital behaviors as part of a broader, integrated social phenomenon. Our study makes several novel contributions to the existing literature:

First, it fills a critical research gap by identifying and systematically categorizing the constituent elements of online moral deviance for the first time. On one hand, within the research themes of *cyber deviance, online deviance*, and *online deviant behavior*, digital behaviors such as internet plagiarism, online trolling, cyberbullying, online harassment, academic misconduct, online cheating, hacking, digital piracy, and internet pornography have often been broadly grouped under these umbrella terms. However, prior literature on these themes has not differentiated these digital behaviors through both moral and legal lenses—this lack of distinction constitutes a major gap in the existing research. Our study excludes digital behaviors that may be viewed as violations of legal standards and instead focuses on identifying and categorizing digital behaviors that, from a moral framework perspective, are seen as violating widely accepted moral expectations within a specific society's dominant culture.

On the other hand, existing research on *cyber deviance, online deviance*, and *online deviant behavior* has predominantly focused on individual types of digital behavior. For instance, studies typically center on one specific form, such as internet plagiarism, online harassment, or online flaming. As Udris (2017) emphasized, researchers have demonstrated a preference for examining specific phenomena rather than adopting an integrated approach to these behavioral patterns. Our study addresses this gap by integrating these independent digital behaviors from a moral framework perspective.

Second, our study is the first to construct a conceptual model of online moral deviance that incorporates a moral explanatory dimension grounded in specific socio-cultural contexts. The integration of digital behaviors identified in prior studies under the themes of *cyber deviance, online deviance*, and *online deviant behavior* is based on three criteria: (1) the moral characteristics of these behaviors, (2) the typical ways they violate widely accepted moral expectations in a given society, and (3) the major harm they cause to online target users. Based on analysis across these dimensions, the identified online behaviors were found to violate moral values such as honesty, fairness, respect, empathy, and responsibility, which are widely accepted within the dominant culture of specific societies. It is important to clarify that the concept of morality in our study is not abstract or universalist but is instead situated within specific cultural and social contexts. Therefore, the construction of our model is also based on culturally and contextually embedded moral conceptions. In our model, moral deviance is evaluated not on the basis of abstract universal norms, but rather through the lens of what has been documented in the existing literature as reflecting what is perceived to be a violation of accepted moral expectations in the dominant culture of a given society.

Third, the findings support the promotion of moral education and the development of healthy online behavior. By identifying, categorizing, and integrating representative online behaviors that violate widely accepted moral expectations within specific social and cultural contexts, this conceptual model can be used to guide future research, policy, and moral education interventions. As discussed in the *Introduction* section, our study provides a practically valuable conceptual model for policymakers, educators, and parents.

It is also worth noting that much of the current research on cyber deviance has focused on adolescents as the primary sample group (e.g., Holt et al., 2012; Lee, 2018; Udris, 2017; Chen et al., 2021; Virgara and Whitten, 2023; Whitten et al., 2024). A few studies have examined adult employees (e.g. Louderback and Antonaccio, 2021). In contrast, our study includes a broader range of populations, such as adolescents, adults, academic researchers, and workplace employees. It highlights that members of these diverse groups are all capable of engaging in morally deviant behaviors in online spaces. This broader inclusion strengthens the generalizability of the study's findings by emphasizing that online moral deviance is not confined to a particular demographic.

To further advance research on online moral deviance, it remains necessary to explore whether behavior-related theories—such as power-control theory, self-control theory, and social cognitive theory—can support investigations into the formation mechanisms and influencing factors of online moral deviance. Additionally, questions concerning how to refine the inclusion criteria for incorporating behaviors into the conceptual model, and how to clearly delineate and clarify the conceptual boundaries among the constituent elements of the model based on those criteria, warrant further reflection and scholarly inquiry. Considering the current research findings and constraints, the present study puts forward an agenda.

- What other representative forms of online deviant behavior, beyond the components proposed in our research, could be incorporated into the conceptual model of online moral deviance?
- How can the inclusion criteria for online deviant behaviors as components or constitutive elements of online moral deviance be refined and improved?
- What are the causes and regulatory mechanisms of online moral deviance?
- Which theories can provide support for exploring and identifying the underlying mechanisms or influencing factors of online moral deviance?
- How to clearly delineate the conceptual boundaries among different types of online deviant behaviors within the conceptual model?
- How can intervention strategies be developed based on the conceptual model of online moral deviance to prevent and reduce the occurrence of such behavioral patterns?

## 5 Limitations

Several limitations of our study should be acknowledged. Firstly, as Bordua (1967) noted that if we were to consider the field of deviant behavior and social control as merely an aggregation of all special studies of norm-violating behavior, a comprehensive summary would be unfeasible. The specific empirical research conducted in any given period substantially reflects societal interest in particular forms of deviance. Consequently, our study's conception of deviance does not encompass the entire spectrum of behaviors ranging from minor antisocial acts to illegal activities.

Regarding the categories of online moral deviance, existing studies have identified various forms of online deviant behavior, including but not limited to Internet trolling, online flaming, cyberbullying, digital piracy, online pornography, and hacking (Udris, 2017). However, our study is not confined to these behaviors alone. Given that behaviors like online pornography, digital piracy, and hacking are generally classified as illegal activities rather than moral transgressions in various cultures, nations, and regions, we excluded these digital behaviors from our analysis. Instead, we focused on digital behaviors that primarily involve moral considerations and have been extensively studied. Nonetheless, despite this focused approach, our study cannot claim to encompass all morally-related online deviant behaviors, particularly as rapid technological advancements continue to produce new forms of online deviance.

Secondly, unlike a systematic review, an integrative review does not necessarily follow predetermined processes for literature selection and data analysis. The present study builds upon prior research on deviance and online deviance, which necessitated the inclusion of representative works beyond the last 5 years, rather than limiting the review to more recent literature. Consequently, our literature selection extends beyond the past 5 years to ensure that critical, representative studies were considered.

Thirdly, “the relativistic criterion of deviation introduced by the new perspective is in keeping with contemporary sociological principles” (Gibbs, 1966, p. 14). This suggests that the standards for defining deviance may differ across cultures, countries, regions, and groups. As a result, the conceptual model of online moral deviance proposed here may not be universally applicable across all cultural contexts. Researchers applying this model must account for cultural, regional, and societal differences and adjust the model accordingly.

Based on the integration of current research findings on various forms of online deviant behavior, the delineation of conceptual boundaries among different types of online deviant behaviors in our conceptual model may not be entirely precise. This is because overlaps, intersections, or inclusion relationships may exist among these behaviors. In particular, as research on the themes of online deviance, cyber deviance, and online deviant behavior continues to advance, the conceptual distinctions between various forms of online deviant behavior will need to be further clarified and refined in future studies.

## 6 Conclusion

The widespread adoption of the Internet has significantly expanded opportunities for morally deviant behaviors in digital spaces. A deeper understanding of online behavioral patterns that violate mainstream societal moral norms and the construction of a conceptual model of these behaviors can help stakeholders better comprehend and address such behaviors. It also facilitates the development of targeted digital moral education initiatives aimed at enhancing individuals' moral cognition and self-regulation abilities, thereby mitigating the potential psychological and physical harm caused by these behaviors. Furthermore, the research findings can provide a scientific basis for policymakers, educators, and technology platforms to optimize strategies for managing online behavior.

The key components of this model include behaviors such as Internet plagiarism, trolling, cyberbullying, online harassment, academic misconduct, online cheating, and flaming. We argue that this model of online moral deviance is not rigid but rather adaptable, allowing for the inclusion, merger, or exclusion of various forms of deviance as needed. For example, behaviors such as spreading rumors and online exclusion can be understood as subsets of cyberbullying. Any digital behavior that meets the established criteria for moral deviance can be incorporated into this evolving conceptual model.

In conclusion, this adaptable model offers a valuable tool for analyzing and addressing online moral deviance. It not only facilitates a better understanding of how these behaviors manifest but also provides a foundation for developing interventions to reduce their prevalence. Future research should continue to refine this model and ensuring that it remains relevant in an ever-evolving digital landscape. Future research should also bring in cross-cultural perspectives if applicable. For example, how might online moral deviance vary across different cultural contexts?

## References

[B1] Alonzo M. Aiken M. (2004). Flaming in electronic communication. Decis. Support Syst. 36, 205–213. 10.1016/S0167-9236(02)00190-2

[B2] Amigud A. (2020). Cheaters on Twitter: an analysis of engagement approaches of contract cheating services. Stud. High. Educ. 45, 692–705. 10.1080/03075079.2018.1564258

[B3] Andersen I. V. (2021). Hostility online: flaming, trolling, and the public debate. First Mon. 26. 10.5210/fm.v26i3.11547

[B4] Ardi N. Ahmad A. Daud N. Ismail N. (2020). Speech act of flaming in twitter status: issues and concerns in the Malaysian context. Asian J. Univ. Educ. 16, 109–121. 10.24191/ajue.v16i4.11961

[B5] Arisanty M. Wiradharma G. (2022). The motivation of flaming perpetrators as cyberbullying behavior in social media. J. Kajian Komun. 10, 215–227. 10.24198/jkk.v10i2.39876

[B6] Armond A. C. V. Gordijn B. Lewis J. Hosseini M. Bodnár J. K. Holm S. (2021). A scoping review of the literature featuring research ethics and research integrity cases. BMC Med. Ethics 22:50. 10.1186/s12910-021-00620-8 33931043 PMC8086087

[B7] Awdry R. (2021). Assignment outsourcing: moving beyond contract cheating. Assess. Eval. High. Educ. 46, 220–235. 10.1080/02602938.2020.1765311

[B8] Bansal A. Sharma S. M. Kumar K. Aggarwal A. Goyal S. Choudhary K.. (2012). Classification of flames in computer mediated communications. arXiv [Preprint]. arXiv:1202.0617. 10.48550/arXiv.1202.0617

[B9] Bawarith R. Basuhail A. Fattouh A. Gamalel-Din S. (2017). E-exam cheating detection system. Int. J. Adv. Comput. Sci. Appl. 8,176–181. 10.14569/IJACSA.2017.080425

[B10] Bell D. J. Loader B. D. Pleace N. Schuler D. (2004). Cyberculture: The Key Concepts. London: Routledge. 10.4324/9780203647059

[B11] Bhat P. (2024). Coping with hate: exploring Indian journalists' responses to online harassment. Journal. Pract. 18, 337–355. 10.1080/17512786.2023.2250761

[B12] Bishop J. (2014). Representations of “trolls” in mass media communication: a review of media-texts and moral panics relating to “internet trolling”. Int. J. Web Based Communities 10, 7–24. 10.1504/IJWBC.2014.058384

[B13] Bordua D. J. (1967). Recent trends: deviant behavior and social control. Ann. Am. Acad. Polit. Soc. Sci. 369, 149–163. 10.1177/000271626736900115

[B14] Brody N. (2021). Bystander intervention in cyberbullying and online harassment: the role of expectancy violations. Int. J. Commun. 15:21. Available online at: https://ijoc.org/index.php/ijoc/article/view/14169/3342

[B15] Buckels E. E. Trapnell P. D. Paulhus D. L. (2014). Trolls just want to have fun. Pers. Individ. Dif. 67, 97–102. 10.1016/j.paid.2014.01.016

[B16] Bussey K. Luo A. Fitzpatrick S. Allison K. (2020). Defending victims of cyberbullying: the role of self-efficacy and moral disengagement. J. Sch. Psychol. 78, 1–12. 10.1016/j.jsp.2019.11.006 32178806

[B17] Celuch M. Latikka R. Oksa R. Oksanen A. (2023). Online harassment and hate among media professionals: reactions to one's own and others' victimization. Journal. Mass Commun. Q. 100, 619–645. 10.1177/10776990221148987

[B18] Celuch M. Oksa R. Savela N. Oksanen A. (2024). Longitudinal effects of cyberbullying at work on well-being and strain: a five-wave survey study. New Media Soc. 26, 3410–3432. 10.1177/14614448221100782

[B19] Chan T. K. H. Cheung C. M. K. Lee Z. W. Y. (2021). Cyberbullying on social networking sites: a literature review and future research directions. Inf. Manag. 58:2. 10.1016/j.im.2020.103411

[B20] Chen G. M. Pain P. Chen V. Y. Mekelburg M. Springer N. Troger F. (2020). ‘You really have to have a thick skin': a cross-cultural perspective on how online harassment influences female journalists. Journalism 21, 877–895. 10.1177/1464884918768500

[B21] Chen J. K. Chang C. W. Wang Z. Wang L. C. Wei H. S. (2021). Cyber deviance among adolescents in Taiwan: prevalence and correlates. Child. Youth Serv. Rev. 126:106042. 10.1016/j.childyouth.2021.106042

[B22] Cho S. Glassner S. (2021). Impacts of low self-control and opportunity structure on cyberbullying developmental trajectories: using a latent class growth analysis. Crime Delinq. 67, 601–628. 10.1177/0011128720950018

[B23] Choi J. Kruis N. Lee J. (2022). Empathy, self-control, and online harassment: a partial test of Agnew's social concern theory. Comput. Human Behav. 136:107402. 10.1016/j.chb.2022.107402

[B24] Choi M. Park H. J. (2023). Korean adolescents' profiles of digital citizenship and its relations to internet ethics: implications for critical digital citizenship education. Cambridge J. Educ. 53, 567–586. 10.1080/0305764X.2023.2191929

[B25] Cioban S. Lazăr A. R. Bacter C. Hatos A. (2021). Adolescent deviance and cyber-deviance. A systematic literature review. Front. Psychol. 12:748006. 10.3389/fpsyg.2021.748006 34712188 PMC8546304

[B26] Clarke R. Lancaster T. (2006). “Eliminating the successor to plagiarism? Identifying the usage of contract cheating sites,” in Proceedings of the 2nd International Plagiarism Conference (Newcastle: JISC Plagiarism Advisory Service).

[B27] Clinard M. B. Meier R. F. (2011). Sociology of Deviant Behavior, 14th Edn. Belmont, CA: Wadsworth Cengage Learning.

[B28] Coles B. A. West M. (2016). Trolling the trolls: online forum users constructions of the nature and properties of trolling. Comput. Human Behav. 60, 233–244. 10.1016/j.chb.2016.02.070

[B29] Cricenti C. Pizzo A. Quaglieri A. Mari E. Cordellieri P. Bonucchi C.. (2022). Did they deserve it? Adolescents' perception of online harassment in a real-case scenario. Int. J. Environ. Res. Public Health. 19:17040. 10.3390/ijerph192417040 36554921 PMC9778851

[B30] Culpeper J. (2011). Impoliteness: Using Language to Cause Offence (Vol. 28). Cambridge: Cambridge University Press. 10.1017/CBO9780511975752

[B31] Czakert J. P. Reif J. Glazer S. Berger R. (2021). Adaptation and psychometric cross-cultural validation of a workplace cyberbullying questionnaire in Spain and Germany. Cyberpsychol. Behav. Soc. Netw. 24, 831–838. 10.1089/cyber.2020.0856 34152860

[B32] Dan O. (1993). Bullying at School: What We Know and What We Can Do. Oxford: Blackwell.

[B33] Dej E. Kilty J. (2024). “Die alone, old, and let the cat eat your face”: anti-feminist backlash and academic cyber-harassment. Fem. Media Stud. 24, 70–86. 10.1080/14680777.2023.2181140

[B34] Dobrovska D. (2017). “Technical student electronic cheating on examination,” in Interactive Collaborative Learning: Proceedings of the 19th ICL Conference-Volume 1 (Springer International Publishing: New York), 525–531. 10.1007/978-3-319-50337-0_49

[B35] Dodds T. Geboers M. Boukes M. (2024). “It became no man's land”: the burden of moderating online harassment in newswork. *Journal. Pract*. 1–18. 10.1080/17512786.2024.2387664

[B36] Dou G. Xiang Y. Sun X. Chen L. (2020). Link between cyberbullying victimization and perpetration among undergraduates: mediating effects of trait anger and moral disengagement. Psychol. Res. Behav. Manag. 13, 1269–1276. 10.2147/PRBM.S286543 33376422 PMC7762765

[B37] Erdur-Baker Ö. (2010). Cyberbullying and its correlation to traditional bullying, gender and frequent and risky usage of internet-mediated communication tools. New Media Soc. 12, 109–125. 10.1177/1461444809341260

[B38] Eret E. Ok A. (2014). Internet plagiarism in higher education: tendencies, triggering factors and reasons among teacher candidates. Assess. Eval. High. Educ. 39, 1002–1016. 10.1080/02602938.2014.880776

[B39] Erikson K. T. (1962). Notes on the sociology of deviance. Soc. Probl. 9, 307–314. 10.1525/sp.1962.9.4.03a00020

[B40] Eslen-Ziya H. Giorgi A. Ahi C. J. (2024). Digital vulnerabilities and online harassment of academics, consequences, and coping strategies: an exploratory analysis. Fem. Media Stud. 24, 1422–1427. 10.1080/14680777.2023.2281268

[B41] Farrington D. P. Zych I. Ttofi M. M. Gaffney H. (2023). Cyberbullying research in Canada: a systematic review of the first 100 empirical studies. Aggress. Violent Behav. 10.1016/j.avb.2022.101811

[B42] Feenstra R. A. Delgado López-Cózar E. Pallarés-Domínguez D. (2021). Research misconduct in the fields of ethics and philosophy: researchers' perceptions in Spain. Sci. Eng. Ethics 27:1. 10.1007/s11948-021-00278-w 33492516

[B43] Francisco S. C. Felmlee D. H. (2022). What did you call me? An analysis of online harassment towards black and latinx women. Race Soc. Probl. 14, 1–13. 10.1007/s12552-021-09330-7

[B44] Gagliardone I. Gal D. Alves T. Martinez G. (2015). Countering Online Hate Speech. Paris: Unesco Publishing.

[B45] Gao L. Liu J. Wang W. Yang J. Wang P. Wang X. (2020). Moral disengagement and adolescents' cyberbullying perpetration: student-student relationship and gender as moderators. Child. Youth Serv. Rev. 116:105119. 10.1016/j.childyouth.2020.105119

[B46] Geng J. Lei L. (2021). Relationship between stressful life events and cyberbullying perpetration: roles of fatalism and self-compassion. Child Abuse Negl. 120:105176. 10.1016/j.chiabu.2021.105176 34217062

[B47] Geng J. Wang P. Zeng P. Liu K. Lei L. (2022). Relationship between honesty-humility and cyberbullying perpetration: a moderated mediation model. J. Interpers. Violence 37, NP14807–NP14829. 10.1177/08862605211016346 33980060

[B48] Gibbs J. P. (1966). Conceptions of deviant behavior: the old and the new. Pac. Sociol. Rev. 9, 9–14. 10.2307/1388302

[B49] Golf-Papez M. Veer E. (2017). Don't feed the trolling: rethinking how online trolling is being defined and combated. J. Mark. Manag. 33, 1336–1354. 10.1080/0267257X.2017.1383298

[B50] Gorman-Smith D. Tolan P. H. Loeber R. Henry D. B. (1998). Relation of family problems to patterns of delinquent involvement among urban youth. J. Abnorm. Child Psychol. 26, 319–333. 10.1023/A:1021995621302 9826291

[B51] Gosse C. Veletsianos G. Hodson J. Houlden S. Dousay T. A. Lowenthal P. R.. (2021). The hidden costs of connectivity: nature and effects of scholars' online harassment, Learn. Media Technol. 46, 264–280. 10.1080/17439884.2021.1878218

[B52] Gray K. L. (2012). Deviant bodies, stigmatized identities, and racist acts: examining the experiences of African-American gamers in Xbox live. New Rev. Hypermedia Multimed. 18, 261–276. 10.1080/13614568.2012.746740

[B53] Gray K. L. Buyukozturk B. Hill Z. G. (2017). Blurring the boundaries: using gamergate to examine “real” and symbolic violence against women in contemporary gaming culture. Sociology Compass 11:e12458. 10.1111/soc4.12458

[B54] Griffiths M. D. (2014). Adolescent trolling in online environments: a brief overview. Educ. Health 32, 85–87. Available online at: https://irep.ntu.ac.uk/id/eprint/25950/1/221474_PubSub2968_Griffiths.pdf

[B55] Hardaker C. (2010). Trolling in asynchronous computer-mediated communication: From user discussions to academic definitions. J. Politeness Res. 6, 215–242. 10.1515/jplr.2010.011

[B56] Hinduja S. Patchin J. W. (2012). Cyberbullying: neither an epidemic nor a rarity. Eur. J. Dev. Psychol. 9, 539–543. 10.1080/17405629.2012.706448

[B57] Holden O. Norris M. Kuhlmeier V. (2021). Academic integrity in online testing: a research review. Front. Educ. 6:639814. 10.3389/feduc.2021.639814

[B58] Holt T. J. Bossler A. M. May D. C. (2012). Low self-control, deviant peer associations, and juvenile cyberdeviance. Am. J. Crim. Justice 37, 378–395. 10.1007/s12103-011-9117-3

[B59] Holton A. E. Bélair-Gagnon V. Bossio D. Molyneux L. (2023). “Not their fault, but their problem”: organizational responses to the online harassment of journalists. Journal. Pract. 17, 205–220. 10.1080/17512786.2021.1946417

[B60] Houlden S. Hodson J. Veletsianos G. Gosse C. Lowenthal P. Dousay T.. (2022). Support for scholars coping with online harassment: an ecological framework. Fem. Media Stud. 22, 1120–1138. 10.1080/14680777.2021.1883086

[B61] Howard R. M. (1999). Standing in the Shadow of Giants: Plagiarists, Authors, Collaborators. Stamford, CT: Ablex.

[B62] Huang C. Zhang Z. Mao B. Yao X. (2023). An overview of artificial intelligence ethics. IEEE Trans. Artif. Intell. 4, 799–819. 10.1109/TAI.2022.3194503

[B63] Huang C. L. Ling C. Yang S. C. (2024). A new form of academic misconduct: the relationship among individual factors, attitudes, experience, and intentions toward Internet plagiarism. Eur. J. Psychol. Educ. 39, 2245–2266. 10.1007/s10212-023-00776-4

[B64] Huang C. L. Yang S. C. Hsieh L. S. (2019). The cyberbullying behavior of Taiwanese adolescents in an online gaming environment. Child. Youth Serv. Rev. 106:104461. 10.1016/j.childyouth.2019.104461

[B65] Ison D. C. (2020). Detection of online contract cheating through stylometry: a pilot study. Online Learn. J. 24, 142–165. 10.24059/olj.v24i2.2096

[B66] Jin C. Wang B. Ji A. Zhao B. (2022). Perceived parental monitoring and online deviant behavior among Chinese adolescents: a moderated mediation model. J. Child Fam. Stud. 31, 2825–2836. 10.1007/s10826-022-02237-w

[B67] Johnson N. A. Cooper R. B. Chin W. W. (2008). The effect of flaming on computer-mediated negotiations. Eur. J. Inf. Syst. 17, 417–434. 10.1057/ejis.2008.22

[B68] Kantola A. Harju A. A. (2023). Tackling the emotional toll together: how journalists address harassment with connective practices. Journalism 24, 494–512. 10.1177/14648849211055293

[B69] Kayany J. M. (1998). Contexts of uninhibited online behavior: flaming in social newsgroups on Usenet. J. Am. Soc. Inf. Sci. 49, 1135–1141.

[B70] Kazim E. Koshiyama A. S. (2021). A high-level overview of AI ethics. Patterns 2:100314. 10.1016/j.patter.2021.100314 34553166 PMC8441585

[B71] Keresztury B. Cser L. (2013). New cheating methods in the electronic teaching era. Procedia Soc. Behav. Sci. 93, 1516–1520. 10.1016/j.sbspro.2013.10.074

[B72] Koirala S. (2020). Female journalists' experience of online harassment: a case study of Nepal. Media Commun. 8, 47–56. 10.17645/mac.v8i1.2541

[B73] Kowalski R. M. Limber S. P. Agatston P. W. (2008). Cyberbullying: The New Moral Frontier. Oxford, UK: Blackwell Publishing Ltd., 10. 10.1002/9780470694176

[B74] Krou M. R. Fong C. J. Hoff M. A. (2021). Achievement motivation and academic dishonesty: a meta-analytic investigation. Educ. Psychol. Rev. 33, 427–458. 10.1007/s10648-020-09557-7

[B75] Lea M. O'Shea T. Fung P. Spears R. (1992). ‘*Flaming' in Computer-mediated communication: Observations, Explanations, Implications*. Hemel Hempstead: Harvester Wheatsheaf.

[B76] Lee B. H. (2018). Explaining cyber deviance among school-aged youth. Child Indic. Res. 11, 563–584. 10.1007/s12187-017-9450-2

[B77] Lee J. (2021). Pathways from childhood bullying victimization to young adult depressive and anxiety symptoms. Child Psychiatry Hum. Dev. 52, 129–140. 10.1007/s10578-020-00997-4 32367195

[B78] Lee N. Y. Park A. (2024). How online harassment affects Korean journalists? The effects of online harassment on the journalists' psychological problems and their intention to leave the profession. Journalism 25, 900–920. 10.1177/14648849231166511

[B79] Lee S. M. Lampe C. Prescott J. J. Schoenebeck S. (2022). “Characteristics of people who engage in online harassing behavior,” *in Conference on Human Factors in Computing Systems—Proceedings* (New York, NY: Association for Computing Machinery (ACM)). 10.1145/3491101.3519812

[B80] Leite Â. Cardoso S. Monteiro A. P. (2023). Dark personality traits and online behaviors: Portuguese versions of cyberstalking, online harassment, flaming and trolling scales. Int. J. Environ. Res. Public Health 20:17. 10.3390/ijerph20126136 37372723 PMC10298694

[B81] Lewis S. C. Zamith R. Coddington M. (2020). Online harassment and its implications for the journalist–audience relationship. Digit. Journal. 8, 1047–1067. 10.1080/21670811.2020.1811743

[B82] Li J. Sidibe A. M. Shen X. Hesketh T. (2019). Incidence, risk factors and psychosomatic symptoms for traditional bullying and cyberbullying in Chinese adolescents. Child. Youth Serv. Rev. 107:1. 10.1016/j.childyouth.2019.104511

[B83] Li M. Hussain S. Barkat S. Bostan H. (2023). Online harassment and trolling of political journalists in Pakistan. Journal. Pract. 19, 1–18. 10.1080/17512786.2023.2259381

[B84] Li W. (2023). Moral disengagement, herd mentality, moral Identity, and empathy in cyberbullying roles. J. Aggress. Maltreat. Trauma 32, 1198–1220. 10.1080/10926771.2023.2222079

[B85] Li W. Peng H. (2022). The impact of strain, constraints, and morality on different cyberbullying roles: a partial test of Agnew's general strain theory. Front. Psychol. 13:980669. 10.3389/fpsyg.2022.980669 36324793 PMC9619098

[B86] Liang H. Jiang H. Zhang C. Zhou H. Zhang B. Tuo A. (2022). How does parent-adolescent conflict and deviant peer affiliation affect cyberbullying: examining the roles of moral disengagement and gender. Psychol. Res. Behav. Manag. 15, 2259–2269. 10.2147/PRBM.S371254 36035808 PMC9409373

[B87] Lin S. Xiao B. (2023). The relationship between paranoid tendencies and cyberbullying among chinese adolescents: the mediating role of moral disengagement. Behav. Sci. 13:102. 10.3390/bs13020102 36829331 PMC9952349

[B88] Lingam R. A. Aripin N. (2017). Comments on fire! classifying flaming comments on youtube videos in Malaysia. J. Komunikasi: Malays. J. Commun. 33, 104–118. 10.17576/JKMJC-2017-3304-07

[B89] Llorent V. J. Diaz-Chaves A. Zych I. Twardowska-Staszek E. Marín-López I. (2021). Bullying and cyberbullying in Spain and Poland, and their relation to social, emotional and moral competencies. School Ment. Health 13, 535–547. 10.1007/s12310-021-09473-3

[B90] Lo Cricchio M. G. García-Poole C. te Brinke L. W. Bianchi D. Menesini E. (2020). Moral disengagement and cyberbullying involvement: a systematic review. Eur. J. Dev. Psychol. 18, 271–311. 10.1080/17405629.2020.1782186

[B91] Louderback E. R. Antonaccio O. (2021). New applications of self-control theory to computer-focused cyber deviance and victimization: a comparison of cognitive and behavioral measures of self-control and test of peer cyber deviance and gender as moderators. Crime Delinq. 67, 366–398. 10.1177/0011128720906116

[B92] Lu S. Luqiu L. R. (2023). When will one help? Understanding audience intervention in online harassment of women journalists. Journal. Pract. 19, 523–541. 10.31219/osf.io/8sy4j

[B93] Luck J. A. Chugh R. Turnbull D. Rytas Pember E. (2022). Glitches and hitches: sessional academic staff viewpoints on academic integrity and academic misconduct. High. Educ. Res. Dev. 41, 1152–1167. 10.1080/07294360.2021.1890697

[B94] Luo A. Bussey K. (2019). The selectivity of moral disengagement in defenders of cyberbullying: contextual moral disengagement. Comput. Human Behav. 93, 318–325. 10.1016/j.chb.2018.12.038

[B95] Luo Y. F. Zhang S. Yang S. C. Huang C. L. (2023). Students' judgments on different cyberbullying incidents: the relationship between moral philosophy and intention to engage. Eur. J. Psychol. Educ. 38, 989–1009. 10.1007/s10212-022-00636-7 40479472 PMC9558036

[B96] Malik M. A. Mahroof A. Ashraf M. A. (2021). Online university students' perceptions on the awareness of, reasons for, and solutions to plagiarism in higher education: the development of the asandp model to combat plagiarism. Appl. Sci. 11:12055. 10.3390/app112412055

[B97] Manuoglu E. Öner-Özkan B. (2022). Sarcastic and deviant trolling in Turkey: associations with dark triad and aggression. Soc. Media Soc. 8:205630512211260. 10.1177/20563051221126053

[B98] March E. Marrington J. (2019). A qualitative analysis of internet trolling. Cyberpsychol. Behav. Soc. Netw. 22, 192–197. 10.1089/cyber.2018.0210 30720370

[B99] McCabe D. L. (1999). Academic dishonesty among high school students. Adolescence 34, 681–687. 10730693

[B100] Memon A. R. Mavrinac M. (2020). Knowledge, attitudes, and practices of plagiarism as reported by participants completing the author AID MOOC on research writing. Sci. Eng. Ethics 26, 1067–1088. 10.1007/s11948-020-00198-1 32067186

[B101] Miller K. C. Lewis S. C. (2022). Journalists, harassment, and emotional labor: the case of women in on-air roles at US local television stations. Journalism 23, 79–97. 10.1177/1464884919899016

[B102] Morissey L. (2010). Trolling is a art: towards a schematic classification of intention in internet trolling. Griffith Working Papers in Pragmatics and Intercultural Communication 2, 75–82. Available online at: https://www.semanticscholar.org/paper/Trolling-is-a-art%3A-Towards-a-schematic-of-intention-Morrissey/85869bcd876abf352783f4e5807eb878e5e5f5ab

[B103] Nadim M. Fladmoe A. (2021). Silencing women? Gender and online harassment. Soc. Sci. Comput. Rev. 39, 245–258. 10.1177/0894439319865518

[B104] Navidinia H. Naznean A. Sourani M. Hekmati N. (2024). Academic dishonesty in virtual assessment during the COVID-19 pandemic: a cross-cultural study. Asia-Pac. Educ. Res. 33, 1489–1499. 10.1007/s40299-024-00829-2

[B105] Newey A. K. Magson N. (2010). “A critical review of the current cyberbullying research: Definitional, theoretical and methodological Issues. Where do we go from here,” in Australian Association for Research in Education International Educational Research Conference. Available online at: http://publications.aare.edu.au/10pap/2521NeweyMagson.pdf *(Accessed October* 20, 2024).

[B106] Noorbehbahani F. Mohammadi A. Aminazadeh M. (2022). A systematic review of research on cheating in online exams from 2010 to 2021. Educ. Inf. Technol. 27, 8413–8460. 10.1007/s10639-022-10927-7 35283658 PMC8898996

[B107] Nwosu L. I. Chukwuere J. E. (2020). The attitude of students towards plagiarism in online learning: a narrative literature review. Gend. Behav. 18, 14675–14688. Available online at: https://www.researchgate.net/profile/Joshua-Chukwuere/publication/343471863_The_attitude_of_students_towards_plagiarism_in_online_learning_A_narrative_literature_review/links/5f2b8ce2299bf13404a5d5ae/The-attitude-of-students-towards-plagiarism-in-online-learning-A-narrative-literature-review.pdf

[B108] Oksanen A. Celuch M. Latikka R. Oksa R. Savela N. (2022). Hate and harassment in academia: the rising concern of the online environment. High. Educ. 84, 541–567. 10.1007/s10734-021-00787-4 34840344 PMC8609255

[B109] Olweus D. Limber S. P. (2018). Some problems with cyberbullying research. Curr. Opin. Psychol. 19, 139–143. 10.1016/j.copsyc.2017.04.012 29279213

[B110] Ortiz S. M. (2020). Trolling as a collective form of harassment: an inductive study of how online users understand trolling. Soc. Media Soc. 6, 1–9. 10.1177/2056305120928512

[B111] Ossa F. C. Jantzer V. Neumayer F. Eppelmann L. Resch F. Kaess M. (2023). Cyberbullying and school bullying are related to additive adverse effects among adolescents. Psychopathology 56, 127–137. 10.1159/000523992 35490676

[B112] Osuchukwu N. P. Udem O. K. Nwosu M. (2022). Plagiarism and copyright among LIS professionals in Nigeria: an assumption or a reality? Afr. J. Libr. Arch. Inf. Sci. 32, 233–244. Available online at: https://ajlais.com/index.php/ajlais/article/view/66/64

[B113] O'Sullivan P. B. Flanagin A. J. (2003). Reconceptualizing “flaming” and other problematic messages. New Media Soc. 5, 69–94. 10.1177/1461444803005001908

[B114] Paakki H. Vepsäläinen H. Salovaara A. (2021). Disruptive online communication: how asymmetric trolling-like response strategies steer conversation off the track. Comput. Support. Coop. Work. 30, 425–461. 10.1007/s10606-021-09397-1

[B115] Paciello M. Tramontano C. Nocentini A. Fida R. Menesini E. (2020). The role of traditional and online moral disengagement on cyberbullying: do externalising problems make any difference? Comput. Hum. Behav. 103, 190–198. 10.1016/j.chb.2019.09.024

[B116] Papacharissi Z. (2004). Democracy online: civility, politeness, and the democratic potential of online political discussion groups. New Media Soc. 6, 259–283. 10.1177/1461444804041444

[B117] Parsons T. (1951). The Social System. New York, NY, US: Free Press.

[B118] Patchin J. W. Hinduja S. (2010). Cyberbullying and Self-Esteem^*^. J. Sch. Health 80, 614–621. 10.1111/j.1746-1561.2010.00548.x 21087257

[B119] Pazhouhi S. (2023). Online and offline bullying/harassment and perceived racial/ethnic discrimination among iranian adolescents. Can. J. Sch. Psychol. 38, 333–348. 10.1177/08295735231188008

[B120] Perren S. Corcoran L. Cowie H. Dehue F. Mc Guckin C. Sevcikova A.. (2012). Tackling cyberbullying: review of empirical evidence regarding successful responses by students, parents, and schools. Int. J. Conflict Violence 6, 283–292. 10.4119/ijcv-2919

[B121] Peters M. A. White E. J. Besley T. Locke K. Redder B. Novak R.. (2021). “Video ethics in educational research involving children: Literature review and critical discussion,” in The Methodology and Philosophy of Collective Writing (London: Routledge), 292–313. 10.4324/9781003171959-18

[B122] Petit J. Li C. Ali K. (2021). Fewer people, more flames: how pre-existing beliefs and volume of negative comments impact online news readers' verbal aggression. Telemat. Inform. 56:101471. 10.1016/j.tele.2020.101471

[B123] Phillips W. (2015). This Is Why We Can't Have Nice Things: Mapping The Relationship Between Online Trolling and Mainstream Culture. Mit Press. Cambridge, MA. 10.7551/mitpress/10288.001.0001

[B124] Plé L. Demangeot C. (2020). Social contagion of online and offline deviant behaviors and its value outcomes: the case of tourism ecosystems. J. Bus. Res. 117, 886–896. 10.1016/j.jbusres.2019.06.002

[B125] Powell A. Henry N. (2015). Digital Harassment and Abuse of Adult Australians: A Summary Report. Australia: Tech and Me Project, RMIT University and La Trobe University. Available online at: https://research.techandme.com.au/wp-content/uploads/REPORT_AustraliansExperiencesofDigitalHarassmentandAbuse.pdf

[B126] Ramim M. M. (2005). “*Towards an understanding and definition of academic misconduct in online learning* environments,” in Conference Proceedings - IEEE Southeastcon (Fort Lauderdale, FL: IEEE).

[B127] Roig M. (2012). Avoiding unethical writing practices. Food Chem. Toxicol. 50, 3385–3387. 10.1016/j.fct.2012.06.043 22750724

[B128] Sammut F. Bezzina M. Scerri J. (2023). Under attack in the cyber battlefield: a scoping review of journalists' experiences of cyberharassment. Journal. Pract. 1–29. 10.1080/17512786.2023.2294290

[B129] Sampaio-Dias S. Silveirinha M. J. Garcez B. Subtil F. Miranda J.. (2024). “Journalists are prepared for critical situations … but we are not prepared for this”: empirical and structural dimensions of gendered online harassment. Journal. Pract. 18, 301–318. 10.1080/17512786.2023.2250755

[B130] Sanfilippo M. R. Fichman P. Yang S. (2018). Multidimensionality of online trolling behaviors. Inf. Soc. 34, 27–39. 10.1080/01972243.2017.1391911

[B131] Scanlon P. M. Neumann D. R. (2002). Internet plagiarism among college students. J. Coll. Stud. Dev. 43, 374–385. Available online at: https://citeseerx.ist.psu.edu/document?repid=rep1&type=pdf&doi=93619e5a4a7dac6591a799732cd7d6a0d2fe635a

[B132] Schoenebeck S. Lampe C. Triệu P. (2023). Online harassment: assessing harms and remedies. Soc. Media Soc. 9:1. 10.1177/20563051231157297

[B133] Selwyn N. (2008). ‘Not necessarily a bad thing …': a study of online plagiarism amongst undergraduate students. Assess. Eval. High. Educ. 33, 465–479. 10.1080/02602930701563104

[B134] Shah S. F. A. Cvetkovic I. Ginossar T. Ullah R. Baber D. Slaughter A. (2024). Online harassment, psychological stressors, and occupational dysfunction among journalists working in a conflict zone. Digit. Journal. 12, 1–18. 10.1080/21670811.2024.2308582

[B135] Shahabuddin S. (2009). Plagiarism in academia. Int. J. Teach. Learn. High. Educ. 21, 353–359. Available online at: https://files.eric.ed.gov/fulltext/EJ909069.pdf

[B136] Siddhpura A. Siddhpura M. (2020). “Plagiarism, contract cheating and other academic misconducts in online engineering education: analysis, detection and prevention strategies” in Proceedings of 2020 IEEE International Conference on Teaching, Assessment, and Learning for Engineering, TALE 2020 (Takamatsu: IEEE). 10.1109/TALE48869.2020.9368311

[B137] Siddiqua A. Gong J. Aksar I. A. (2023). Twitter trolling of Pakistani female journalists: a patriarchal society glance. Media Cult. Soc. 45, 1303–1314. 10.1177/01634437231168306

[B138] Sisti D. A. (2007). How do high school students justify Internet plagiarism? Ethics Behav. 17, 215–231. 10.1080/10508420701519163

[B139] Smith P. K. Mahdavi J. Carvalho M. Fisher S. Russell S. Tippett N. (2008). Cyberbullying: its nature and impact in secondary school pupils. J. Child Psychol. Psychiatry Allied Discip. 49, 376–385. 10.1111/j.1469-7610.2007.01846.x 18363945

[B140] Snyder H. (2019). Literature review as a research methodology: an overview and guidelines. J. Bus. Res. 104, 333–339. 10.1016/j.jbusres.2019.07.039

[B141] Sorea D. Repanovici A. (2020). Project-based learning and its contribution to avoid plagiarism of university students. Invest. Bibliotecol. 34, 155–178. 10.22201/iibi.24488321xe.2020.85.58241

[B142] Spaccatini F. Pacilli M. G. Pagliaro S. Giovannelli I. (2023). Victim blaming 2.0: blaming sexualized victims of online harassment lowers bystanders' helping intentions. Curr. Psychol. 42, 19054–19064. 10.1007/s12144-022-02884-8

[B143] Srikanth M. Asmatulu R. (2014). Modern cheating techniques, their adverse effects on engineering education and preventions. Int. J. Mech. Eng. Educ. 42, 129–140. 10.7227/IJMEE.0005

[B144] Stephens J. M. Bertram Gallant T. (2024). Enhancing moral sensitivity in the aftermath of academic misconduct: results from a quasi-experimental field study. J. Moral Educ. 53, 592–607. 10.1080/03057240.2023.2268298

[B145] Stephens J. M. Watson P. W. S. J. Alansari M. Lee G. Turnbull S. M. (2021). Can online academic integrity instruction affect university students' perceptions of and engagement in academic dishonesty? Results from a natural experiment in New Zealand. Front. Psychol. 12:569133. 10.3389/fpsyg.2021.569133 33679506 PMC7928306

[B146] Stephens J. M. Young M. F. Calabrese T. (2007). Does moral judgment go offline when students are online? A comparative analysis of undergraduates' beliefs and behaviors related to conventional and digital cheating. Ethics Behav. 17, 233–254. 10.1080/10508420701519197

[B147] Stevens F. Nurse J. R. C. Arief B. (2021). Cyber stalking, cyber harassment, and adult mental health: a systematic review. Cyberpsychol. Behav. Soc. Netw. 24, 367–376. 10.1089/cyber.2020.0253 33181026

[B148] Stockemer D. Reidy T. (2024). Academic misconduct, fake authorship letters, cyber fraud: evidence from the international political science review. Learn. Publ. 37. 10.1002/leap.1587

[B149] Summers R. W. (2017). “Social psychology: how other people influence our thoughts and actions. Vol. 1” in Social Psychology: How Other People Influence Our Thoughts and Actions. 10.5040/9798216015956

[B150] Sureda Garcia I. López Penádes R. Rodríguez Rodríguez R. Sureda Negre J. (2020). Cyberbullying and Internet addiction in gifted and nongifted teenagers. Gift. Child Q. 64, 192–203. 10.1177/0016986220919338

[B151] Takano M. Taka F. Ogiue C. Nagata N. (2024). Online harassment of Japanese celebrities and influencers. Front. Psychol. 15:1386146. 10.3389/fpsyg.2024.1386146 38686089 PMC11057462

[B152] Tandoc E. C. Sagun K. K. Alvarez K. P. (2023). The digitization of harassment: women journalists' experiences with online harassment in the Philippines. Journal. Pract. 17, 1198–1213. 10.1080/17512786.2021.1981774

[B153] Teng Z. Nie Q. Zhu Z. Guo C. (2020). Violent video game exposure and (Cyber)bullying perpetration among Chinese youth: the moderating role of trait aggression and moral identity. Comput. Hum. Behav. 104:106193. 10.1016/j.chb.2019.106193

[B154] Thacker S. Griffiths M. D. (2012). An exploratory study of trolling in online video gaming. Int. J. Cyber Behav. Psychol. Learn. 2, 17–33. 10.4018/ijcbpl.2012100102

[B155] Thompsen P. A. (1993). “A social influence model of flaming in computer-mediated communication,” in Paper presented at the 64th Annual Meeting of the Western States Communication Association, Albuquerque, NM, 12–16 February. ERIC (ED355572).

[B156] Torraco R. J. (2005). Writing integrative literature reviews: guidelines and examples. Hum. Resour. Dev. Rev. 4, 356–367. 10.1177/1534484305278283

[B157] Turnage A. K. (2007). Email flaming behaviors and organizational conflict. J. Comput.-Mediat. Commun. 13, 43–59. 10.1111/j.1083-6101.2007.00385.x

[B158] Udris R. (2017). Psychological and social factors as predictors of online and offline deviant behavior among Japanese adolescents. Deviant Behav. 38, 792–809. 10.1080/01639625.2016.1197689

[B159] Uwalaka T. Amadi A. F. Nwala B. Wokoro P. (2023). Online harassment of journalists in Nigeria: audience motivations and solutions. Media Int. Aust. 194, 115–130. 10.1177/1329878X231206840

[B160] Uwalaka T. Amadi F. (2023). Beyond “online notice-me”: analysing online harassment experiences of journalists in Nigeria. Journal. Stud. 24, 1937–1956. 10.1080/1461670X.2023.2260499

[B161] Vale M. Pereira F. Spitzberg B. H. Matos M. (2022). Cyber-harassment victimization of Portuguese adolescents: a lifestyle-routine activities theory approach. Behav. Sci. Law 40, 604–618. 10.1002/bsl.2596 36102898

[B162] van Baak C. Maher C. A. Protas M. E. Hayes B. E. (2023). Victims and perpetrators of cyber harassment: the role of power and control and the use of techniques of neutralization. Deviant Behav. 44, 690–707. 10.1080/01639625.2022.2088317

[B163] Vera-Gray F. (2017). Talk about a cunt with too much idle time': trolling feminist research. Fem. Rev. 115, 61–78. 10.1057/s41305-017-0038-y

[B164] Virgara J. L. Whitten T. (2023). A systematic literature review of the longitudinal risk factors associated with juvenile cyber-deviance. Comput. Hum. Behav. 141:107613. 10.1016/j.chb.2022.107613

[B165] Waechter N. Meschik M. (2023). Peer socialization of male adolescents in digital games: achievement, competition, and harassment. Communications 48, 457–481. 10.1515/commun-2021-0079

[B166] Wagner A. (2022). Tolerating the trolls? Gendered perceptions of online harassment of politicians in Canada. Fem. Media Stud. 22, 32–47. 10.1080/14680777.2020.1749691

[B167] Walker M. Townley C. (2012). Contract cheating: a new challenge for academic honesty? J. Acad. Ethics 10, 27–44. 10.1007/s10805-012-9150-y

[B168] Wang L. Ngai S. S. Y. (2021). Understanding the effects of personal factors and situational factors for adolescent cyberbullying perpetration: the roles of internal states and parental mediation. J. Adolesc. 89, 28–40. 10.1016/j.adolescence.2021.03.006 33845339

[B169] Wang L. Ngai S. S. yum. (2020). The effects of anonymity, invisibility, asynchrony, and moral disengagement on cyberbullying perpetration among school-aged children in China. Child. Youth Serv. Rev. 119:105613. 10.1016/j.childyouth.2020.105613

[B170] Wang Y. Xu Z. (2021). Statistical analysis for contract cheating in Chinese universities. Mathematics 9:1684. 10.3390/math9141684

[B171] Whitten T. Cale J. Brewer R. Logos K. Holt T. J. Goldsmith A. (2024). Exploring the role of self-control across distinct patterns of cyber-deviance in emerging adolescence. Int. J. Offender Ther. Comp. Criminol. 1–20. 10.1177/0306624X231220011 38178553 PMC13310271

[B172] Wilson N. C. Seigfried-Spellar K. C. (2023). Cybervictimization, social, and financial strains influence internet trolling behaviors: a general strain theory perspective. Soc. Sci. Comput. Rev. 41, 967–982. 10.1177/08944393211065868

[B173] Wu B. Li F. Zhou L. Liu M. Geng F. (2022). Are mindful people less involved in online trolling? A moderated mediation model of perceived social media fatigue and moral disengagement. Aggress. Behav. 48, 309–318. 10.1002/ab.22013 34897702

[B174] Xu W. Zheng S. (2022). Childhood emotional abuse and cyberbullying perpetration among Chinese university students: the chain mediating effects of self-esteem and problematic social media use. Front. Psychol. 13:1036128. 10.3389/fpsyg.2022.1036128 36533046 PMC9751917

[B175] Xue J. Hu R. Chai L. Han Z. and Sun I. Y. (2022). Examining the prevalence and risk factors of school bullying perpetration among chinese children and adolescents. Front. Psychol. 13:720149. 10.3389/fpsyg.2022.720149 35369167 PMC8967130

[B176] Yang J. Li S. Gao L. Wang X. (2022b). Longitudinal associations among peer pressure, moral disengagement and cyberbullying perpetration in adolescents. Comput. Hum. Behav. 137:107420. 10.1016/j.chb.2022.107420

[B177] Yang J. Li W. Gao L. Wang X. (2022a). How is trait anger related to adolescents' cyberbullying perpetration? A moderated mediation analysis. J. Interpers. Violence 37, NP6633–NP6654. 10.1177/0886260520967129 33084460

[B178] Yazici S. Yildiz Durak H. Aksu Dünya B. Sentürk B. (2023). Online versus face-to-face cheating: the prevalence of cheating behaviours during the pandemic compared to the pre-pandemic among Turkish University students. J. Comput. Assist. Learn. 39, 231–254. 10.1111/jcal.12743

[B179] Yeon Lee N. Park A. (2024). Unraveling the digital threat: exploring the impact of online harassment on South Korean journalists' professional roles. Journal. Mass Commun. Q. 101, 529–551. 10.1177/10776990231217448

[B180] Young J. R. (2001). The Cat-and-Mouse Game of Plagiarism Detection. Washington, DC: The Chronicle of Higher Education.

[B181] Yudes C. Rey L. Extremera N. (2022). The moderating effect of emotional intelligence on problematic internet use and cyberbullying perpetration among adolescents: gender differences. Psychol. Rep. 125, 2902–2921. 10.1177/00332941211031792 34240633

[B182] Zayed H. (2023). “We are not cheating. *We are helping each other out:” digital collective cheating in secondary education. Learn. Media Technol. 49, 1–19*. 10.1080/17439884.2023.2222621

[B183] Zhan J. Yang Y. Lian R. (2022). The relationship between cyberbullying victimization and cyberbullying perpetration: the role of social responsibility. Front. Psychiatry. 13:995937. 10.3389/fpsyt.2022.995937 36159922 PMC9500317

[B184] Zhang H. Sun X. Chen L. Yang H. Wang Y. (2020). The mediation role of moral personality between childhood psychological abuse and cyberbullying perpetration attitudes of college students. Front. Psychol. 11:1215. 10.3389/fpsyg.2020.01215 32581973 PMC7289981

[B185] Zhang L. Amos C. Pentina I. (2024). Interplay of rationality and morality in using ChatGPT for academic misconduct. Behav. Inf. Technol. 44:1. 10.1080/0144929X.2024.2325023

[B186] Zhang S. (2021). From flaming to incited crime: recognising cyberbullying on Chinese wechat account. Int. J. Semiotics Law 34, 1093–1116. 10.1007/s11196-020-09790-x

[B187] Zhao L. Yu J. (2021). A meta-analytic review of moral disengagement and cyberbullying. Front. Psychol. 12:681299. 10.3389/fpsyg.2021.681299 34916984 PMC8669765

[B188] Zhou Y. Liu W. Lee C. Xu B. Sun I. (2024). Traditional social learning predicts cyber deviance? Exploring the offending versatility thesis in social learning theory. Behav. Sci. Law 42, 417–434. 10.1002/bsl.2664 38769070

[B189] Zviyita I. Mare A. (2024). Same threats, different platforms? Female journalists' experiences of online gender-based violence in selected newsrooms in Namibia. Journalism 25, 779–799. 10.1177/14648849231183815

[B190] Zych I. Gómez-Ortiz O. Fernandez Touceda L. Nasaescu E. Llorent V. J. (2020). Parental moral disengagement induction as a predictor of bullying and cyberbullying: mediation by children's moral disengagement, moral emotions, and validation of a questionnaire. Child Indic. Res. 13, 1065–1083. 10.1007/s12187-019-09670-2

